# Structural landscapes of PPI interfaces

**DOI:** 10.1093/bib/bbac165

**Published:** 2022-06-02

**Authors:** Carlos H M Rodrigues, Douglas E V Pires, Tom L Blundell, David B Ascher

**Affiliations:** Computational Biology and Clinical Informatics, Baker Heart and Diabetes Institute, Melbourne, Victoria; Systems and Computational Biology, Bio21 Institute, University of Melbourne, Melbourne, Victoria; School of Chemistry and Molecular Biosciences, Bio21 Institute, University of Queensland, Brisbane, Victoria; Computational Biology and Clinical Informatics, Baker Heart and Diabetes Institute, Melbourne, Victoria; Systems and Computational Biology, Bio21 Institute, University of Melbourne, Melbourne, Victoria; School of Computing and Information Systems, University of Melbourne, Melbourne, Victoria; Department of Biochemistry, University of Cambridge, Cambridge, UK; Computational Biology and Clinical Informatics, Baker Heart and Diabetes Institute, Melbourne, Victoria; Systems and Computational Biology, Bio21 Institute, University of Melbourne, Melbourne, Victoria; School of Chemistry and Molecular Biosciences, Bio21 Institute, University of Queensland, Brisbane, Victoria; Department of Biochemistry, University of Cambridge, Cambridge, UK

**Keywords:** protein–protein interface, structural biology, protein binding site, drug design

## Abstract

Proteins are capable of highly specific interactions and are responsible for a wide range of functions, making them attractive in the pursuit of new therapeutic options. Previous studies focusing on overall geometry of protein–protein interfaces, however, concluded that PPI interfaces were generally flat. More recently, this idea has been challenged by their structural and thermodynamic characterisation, suggesting the existence of concave binding sites that are closer in character to traditional small-molecule binding sites, rather than exhibiting complete flatness. Here, we present a large-scale analysis of binding geometry and physicochemical properties of all protein–protein interfaces available in the Protein Data Bank. In this review, we provide a comprehensive overview of the protein–protein interface landscape, including evidence that even for overall larger, more flat interfaces that utilize discontinuous interacting regions, small and potentially druggable pockets are utilized at binding sites.

## Introduction

Proteins are involved in most fundamental biological processes, including cell proliferation [1], signalling [2], host-pathogen interactions [3] and transport [4], via tightly coordinated and complex networks of interactions. Each protein will often interact through specific regions on their surface with several different protein partners. Given protein size and diversity, in humans, the proteome is estimated to be ~20 000, while the interactome over 650 000 [5], with protein–protein interactions (PPIs) long been considered to offer a highly selective and tunable way to modulate protein activities and pathways.

Originally, interacting interface regions were considered to be large, hydrophobic, flat and featureless [6], leading to their characterisation as poor targets for the development of small molecule modulators. However, recent structural and thermodynamic characterisation [7] has allowed the classification of PPIs based on the nature of interacting partners, and further suggested that binding pockets at the interface may play important roles in molecular recognition and binding. Due to the lack of understanding and complexity of PPI interface regions, however, this remains a challenging area.

While large compilations of PPI networks are important to elucidate which proteins interact with each other, they lack in-depth information of how those interactions occur. Despite a relatively small proportion of the interactome being covered by structural data, advances in experimental structure resolution and application of structural bioinformatics [8–10] add promising contributions to a more complete and broad structural characterisation of PPI interactions.

Here we report the results of a large-scale analysis for the structural landscapes of PPI interfaces based on 3D structures available in the Protein Data Bank (PDB) [11]. We investigate a range of geometric and physicochemical properties of over 55 000 PPI interfaces, including planarity, shape complementary, secondary structure content, solvent accessibility, use of concavity and identification of hotspots, across different classes of interfaces, and discussed implications for druggability.

## Results

### Protein–protein interface properties

Analysis of the interface segmentation distribution of PPI interfaces within the PDB revealed that having up to five interface segments were the most prevalent, accounting for 70% of interfaces (Figure S1 and Table S1, see Supplementary Data available online at https://academic.oup.com/bib), with interactions involving peptides being predominantly single segmented. This allowed us to categorize the interfaces as either single (continuous) or multi-segmented (discontinuous). Figure S2 (see Supplementary Data available online at https://academic.oup.com/bib) shows the distribution of planarity for interfaces of different segmentations and types. Single segmented interfaces were significantly more planar than multi-segmented ones (Table S2, see Supplementary Data available online at https://academic.oup.com/bib) and, while there was no significant difference in segmentation between peptide-type interfaces. Non-identical pairs were significantly more planar than identical pairs with symmetric and non-symmetric interfaces. The former was the most planar among all interface types (Table S3, see Supplementary Data available online at https://academic.oup.com/bib).

Single and multi-segmented interfaces were also largely composed of residues in loops and α-helices in their core and periphery regions (Figures S3 and S4 and Tables S4 and S5, see Supplementary Data available online at https://academic.oup.com/bib). Loop residues dominated on average at smaller sides of single segmented interfaces, and on both sides of multi-segmented interfaces, while β-sheet residues were significantly less prevalent in all interfaces. However, α-helix dominated in the interface cores of multi-segmented interfaces and the larger sides of single segmented interfaces, but not in the smaller sides of single segmented interfaces. Loops were significantly more present in the interface peripheries of all segmentations of interfaces than α-helix, which in turn were significantly more present than β-sheet residues. With respect to secondary structure use by interface types, loops were more prevalent at identical non-symmetrical interfaces than α-helix, whereas there were no significant differences in α-helix and loop usage in identical symmetric interfaces. Peptides had significantly more loops than other interfaces; however, while the enzyme’s interface regions of Enzyme-Peptide interfaces tended to be formed of loops, the protein interface regions of Protein-peptide interfaces were significantly more helical than unstructured. In the interface core, however, for peptides of Protein-peptide interfaces, α-helix made up a greater proportion of interface cores than all other types of interfaces, and helices were significantly more present in identical symmetric core residues than loops. Loops were significantly more present in the interface peripheries of all interface types, followed by α-helix and β-sheets.

With respect to Normalized Interface Packing (NIP), single segmented interfaces were significantly more well-packed than multi-segmented interfaces (Figure S5 and Table S6, see Supplementary Data available online at https://academic.oup.com/bib). Peptidic interfaces were the most well packed, followed by identical pairs with non-symmetric interfaces and non-identical pairs, which did not differ significantly in packing, and identical pairs with symmetric interfaces (Table S7, see Supplementary Data available online at https://academic.oup.com/bib). Similar to NIP, Normalized Shape correlation (NSc) was significantly higher in single segmented interfaces than in multi-segmented interfaces (Figure S6 and Table S8, see Supplementary Data available online at https://academic.oup.com/bib). Peptidic interfaces were the most complementary; however, enzyme-peptide interfaces had significantly higher NSc values than protein-peptide ones. Identical pairs with symmetric interfaces were the least complementary and non-identical pairs and identical pairs with non-symmetric interfaces were not significantly different from each other (Table S9, see Supplementary Data available online at https://academic.oup.com/bib).

The average buried surface area (BSA) was significantly higher for multi-segmented interfaces than single segmented interfaces, by over 1000 Å [2] (Figure S7, see Supplementary Data available online at https://academic.oup.com/bib). Single segmented interfaces used significantly greater proportions of interface core residues on their larger sides than either side of multi-segmented interfaces (Tables S10 and S11, see Supplementary Data available online at https://academic.oup.com/bib). However, they utilized a significantly smaller proportion of interface core residues per interface on the smaller side of the interface than multi-segmented interfaces, which differ significantly between smaller and larger side (Figures S8 and S9 and Tables S12 and S13, see Supplementary Data available online at https://academic.oup.com/bib).

Looking at the intermolecular interactions per 100 Å [2] BSA revealed interesting differences between the types of interfaces. Figures S10 and S11 and Tables S14–S45 (see Supplementary Data available online at https://academic.oup.com/bib) show distributions of use of non-covalent contacts for PPI interfaces in the dataset, by interface segmentation and interface type, respectively. Single segmented interfaces were significantly enriched in VdW, hydrogen/polar, atom–ring interactions compared with interfaces with multiple segments, which showed to have significantly more ionic, hydrophobic, carbonyl, amide-ring and amide–amide interactions. With respect to types of interfaces, individual interaction types showed different variations. For some interface types, numbers of interactions per 100 Å [2] BSA matched those elucidated from analyzing interactions by interface segmentation alone, such as peptidic interfaces making greater use of VDW clash, proximal, hydrogen/polar bonding, weak hydrogen/polar bonding, hydrophobic, carbonyl, atom–ring interactions. However, in other cases, variations between use of interactions were more interface type-dependent than segmentation-dependent. For example, there has been significantly more use of amide–amide interactions by identical pairs with non-symmetric interfaces than any other interface type, with the exception of Protein-peptide interfaces, which made use of significantly fewer ionic interactions. No other differences among other interface types was observed. Identical pairs with symmetric interfaces consistently made significantly lower or similar use of non-covalent interactions, with the exceptions of amide–amide, Carbon–PI and ionic interactions, compared with all other interface types.

### Concavity across interfaces

**Figure 1:**
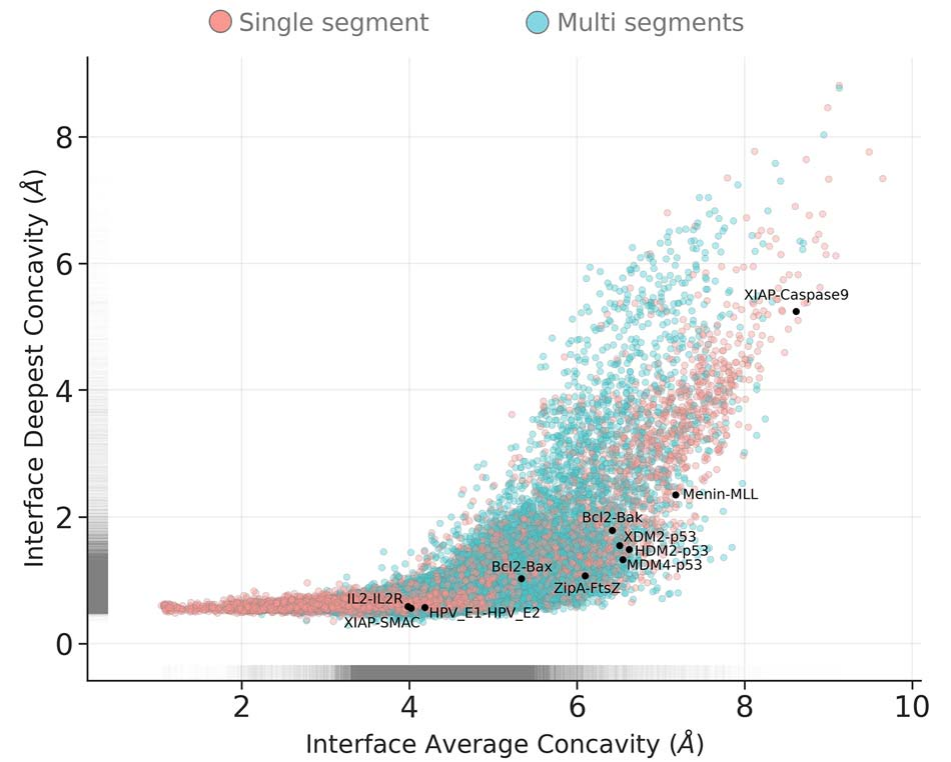
Point and 2D density distributions of occupation of concavity at PPI interfaces, on average and at deepest point. Each point represents the smaller side of one interface from the non-redundant set of non-overlapping PPI interfaces. Concavity is as measured by Ghecom, representing the smallest spherical probe size that was able to enter a space around the partner protein’s surface (where smaller values represent deeper binding). Interfaces are coloured by segmentation, and PPI interfaces from the 2P2I dataset for which small-molecule inhibitors have been developed are overlaid as black points and labelled.

**Figure 2:**
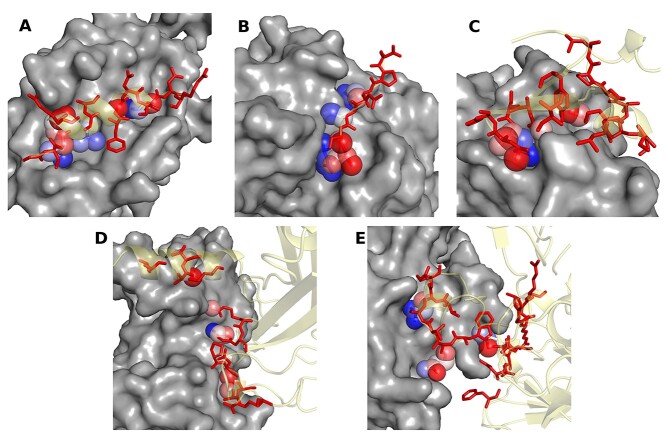
Structural examples of use of concavity for different types of PPI interfaces. Larger molecules are shown in grey surface representation as a ‘receptor’ and the other interacting molecule is shown in translucent yellow as a ‘ligand’. Residues at the interface are shown as red sticks. Atoms occupying deeper concavities are shown as spheres and coloured in a rainbow scale from deepest (blue) to more ‘shallow’ atoms (red). Panels (**A**) and (**B**) show pairs of interactions for protein–peptide (PBD: 2LP8) and enzyme–peptide (PDB: 8PCH) interactions, respectively. Panel (**C**) summarizes an interaction between two different proteins (PDB: 1OQD), namely Heteropair. Panels (**D**) and (**E**) represent associations between two nearly identical proteins (over 95% identity), using different interface residues (PDB: 1CZY) and nearly identical residues on each side of the interface (PDB: 3FPC), respectively.

Concave geometry of protein surfaces is implicated in the formation of surface regions suitable for the binding of small, potentially drug-like, molecules. The majority of observations indicated that both single and multi-segmented interfaces made use of concavities over the whole interface surface; however, single segmented interfaces were bound significantly deeper on average, binding at a ‘groove’ magnitude of concavity (Figures 1 and S12 and Tables S46 and S47, see Supplementary Data available online at https://academic.oup.com/bib). By comparison, small-molecule natural product ligands occupy concavities of less than 5 Å with 60–95% of their atoms [12] (measured per atom, rather than summarized by deepest value per residue). In addition, analysis of a subset of PPI interfaces with known small molecule orthosteric modulators, extracted from 2P2I, showed the majority of interfaces having atoms occupying deep concavities (<4 Å), except for the XIAP-Caspase-9 complex, which binds to a larger and flatter region. Figure 2 shows structural examples of PPI interfaces in the context of their concavity utilization.

**Figure 3:**
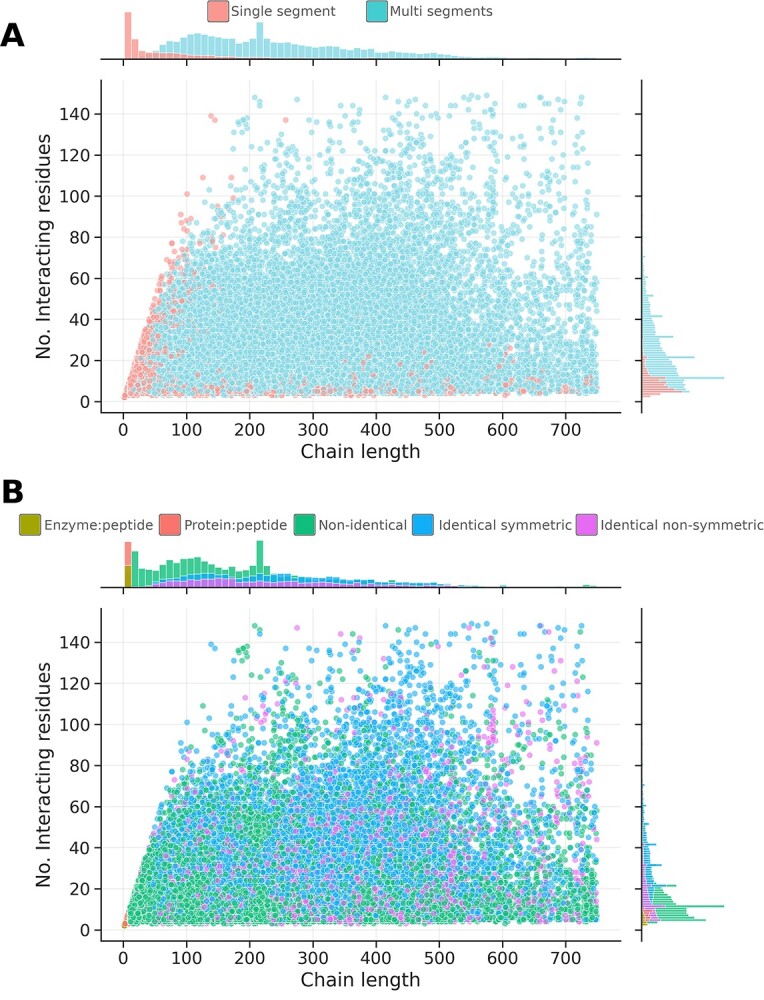
2D density distributions showing interface classifications by chain length and size of interacting surfaces. Density distributions are shown at a single density level for interfaces by (**A**) segmentation and (**B**) interface type.

The importance of concavity on average and at the deepest level varied as the protein molecule size and interface size of the protomer increased (Figure S13, see Supplementary Data available online at https://academic.oup.com/bib) (*R* = 0.32, *P*-value < 0.05). Both single and multi-segmented interfaces exhibited outliers with very large chain lengths. Single segmented interfaces also utilized significantly fewer interacting residues than multi-segmented interfaces (Figure 3A and Table S48, see Supplementary Data available online at https://academic.oup.com/bib), while each globular interface type was significantly different in number of interacting residues from one another (Table S49, see Supplementary Data available online at https://academic.oup.com/bib). No significant difference in the chain length for the two types of peptidic interfaces was observed, neither between identical pairs with symmetric and non-symmetric interfaces (Figure 3B and Tables S50 and S51, see Supplementary Data available online at https://academic.oup.com/bib). Notably, identical pairs with symmetric interfaces used significantly more residues than all the other types of interfaces.

Inspecting averaged concavity values showed that smaller protomers with smaller interfaces were more likely to utilize concavity on average (Figure S13 and Tables S46 and S47, see Supplementary Data available online at https://academic.oup.com/bib). As protomer length increased, interfaces became overall flatter regardless of the number of interacting residues. With respect to deepest concavity utilized at interfaces, deep concavities (<4 Å) were utilized by at least part of the interface for a majority of observations. However, interface deepest concavity tended to take less concave values for longer protomers with fewer interacting residues. Some exceptions to this trend were represented by longer protomers using deep concavities at their deepest, although the interacting region of these two large chains resembles more a peptidic interface.

### Exploring use of concavity

Looking more closely, we analyzed how concavity at interfaces was used by individual residues. Residue utilization of concavity, how well the residues of one side of each interface make use of the (sub-)pockets available to them on the partner protein, varied with the nearby formation of concavity on the binding partner protein (Figures S14 and S15, see Supplementary Data available online at https://academic.oup.com/bib). Here, single and multi-segmented interfaces made use of concavity in both the core and periphery. For multi-segment/globular interface categories, residues in the interface core were observed in bimodal distributions; a mode where the residue is bound deeply and using local concavity, and a mode where the residue is bound with varying degrees of local concavity on the partner chain. Multi-segment interfaces utilising discontinuous binding regions were not only larger than single segmented interfaces, but also less well packed and less complementary in shape compared with single segment interfaces. These observations suggest that single interacting segments make tight, selective interactions with their globular partner proteins, compared with looser interfaces in larger multi-segmented complexes. Interface core residues showed deepest average use of concavity for peptidic interfaces, and peptide interface periphery residues occupied deeper concavities than identical pairs with symmetric and non-symmetric interface core residues, which did not differ significantly.

**Figure 4:**
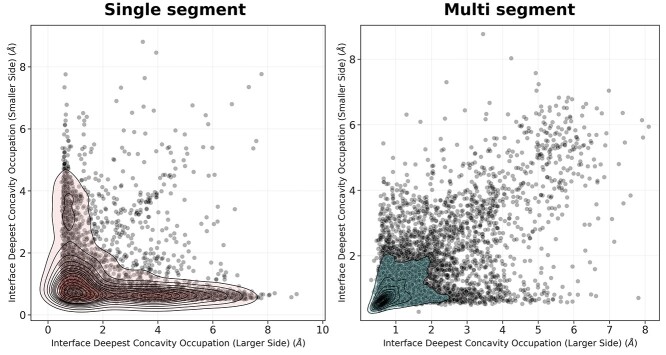
Point and 2D density distributions of deepest concavity occupation on the larger and smaller sides of PPI interfaces. Concavity is as measured by Ghecom, representing the smallest spherical probe size that was able to enter a space sound the partner protein’s surface (where smaller values represent deeper binding). Density distributions are coloured by interface segmentation.

The large proportion of interfaces that at their deepest occupied deep concavities (Figures S14 and S15, see Supplementary Data available online at https://academic.oup.com/bib) raised the hypothesis that both surfaces of PPI interfaces provide ‘anchoring’ points for one another. Analysis of interfaces revealed that an ‘interlocking’ phenomenon, where deep concavity utilized in the 0.5 Å–2 Å range was complemented by reciprocal concavity use on the other side of the interface, existed in a greater proportion for multi-segmented/globular interfaces, than for single segmented/peptidic interfaces (Figures 4 and S16, see Supplementary Data available online at https://academic.oup.com/bib). Helix residues bound significantly deeper than loop and sheet residues in single segmented interfaces given the same solvent accessibility, for multi-segmented interfaces helices and sheets bound significantly deeper than loops; however, they were not significantly different from each other (Figures S17 and S18 and Tables S52 and S53, see Supplementary Data available online at https://academic.oup.com/bib).

### Energetic hot spots

Hotspot density in different interface segmentations and types was calculated using mCSM-PPI to identify the number of hotspots per 100 Å [2] BSA (Figure S19, see Supplementary Data available online at https://academic.oup.com/bib). Single segment interfaces used significantly more hotspots per 100 Å [2] BSA than multi-segmented interfaces. Interfaces involving peptides had the highest densities of hotspots and were significantly different between the two classes (enzyme–peptide and protein–peptide interactions) (Tables S54 and S55, see Supplementary Data available online at https://academic.oup.com/bib). For interactions involving globular proteins, identical pairs with symmetric interactions used significantly more hotspots per 100 Å [2] BSA than the other two classes and identical pairs with non-symmetric interfaces utilized significantly fewer hotspots per 100 Å [2] BSA than any other interface type. Figures S20 and S21 (see Supplementary Data available online at https://academic.oup.com/bib) illustrate the relationship between residue use of concavity, solvent accessibility and energetic importance for each type of interface in the dataset. Overall, for residues originating from the more deeply bound sides of interfaces, there was no significant correlation between residue occupation of concavity and energetic importance (Pearson correlation coefficient *R* = −0.05). When separated by solvent accessibility, the correlations were *R* = 0.23 for interface core residues and *R* = 0.02 for peripheral residues. Correlations of hotspots with use of concavity ranged from −0.04 to 0.25 for all interface types and environments (Figure S21, see Supplementary Data available online at https://academic.oup.com/bib).

### Clustering of orthosteric sub-pockets on PPI interfaces

The anchor hypothesis of interaction proposes that initial, fast recognition between protomers is mediated by residues, usually from the smaller interacting partner, that bury a large portion (>100 Å [2]) of surface area and adopt the same rotameric states when bound and unbound. We explored this concept using concavity as a metric for determining anchoring residues, in addition to solvent accessibility, which we define here as ‘enclosed’ residues. Looking at the numbers of enclosed residues present in PPI interfaces (Figure S22, see Supplementary Data available online at https://academic.oup.com/bib) showed that around 80% of PPI interfaces had at least one enclosed residue. Enzyme-peptide interfaces exhibited the largest proportion of interfaces with at least one enclosed residue (93%), followed by Protein-Peptides (90%), identical pairs with symmetric interface (88%), identical pairs with non-symmetric interface (76%) and non-identical pairs (75%).

**Figure 5:**
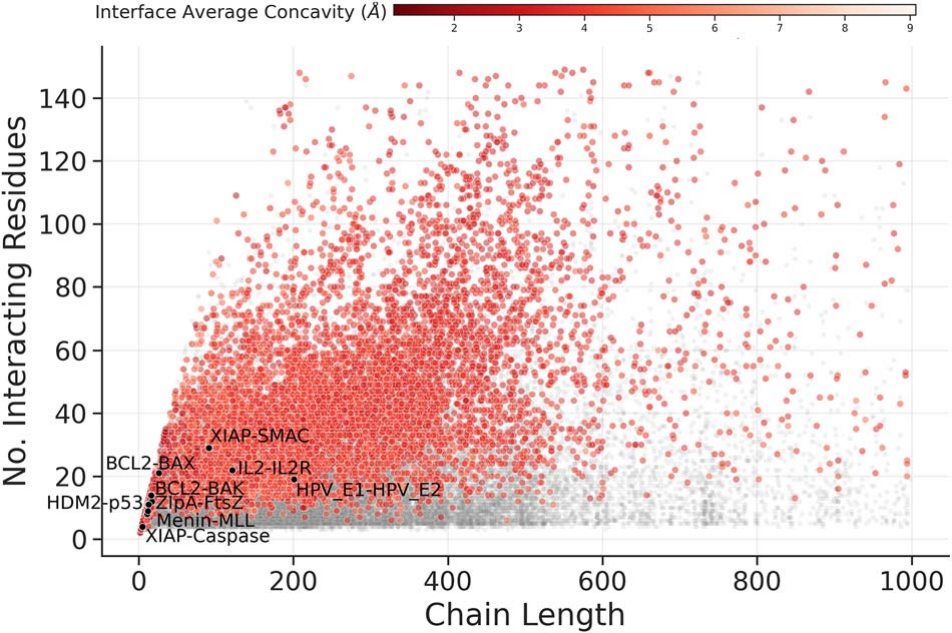
Elucidating potential orthosteric binding pockets utilized by PPI protein partners, by clustering deeply bound, solvent inaccessible interface residues. The distribution of protein partner chain length as compared with binding site size is shown as grey points overlaid with coloured circles representing interfaces for which clusters of enclosed residues were found. Interfaces from the 2P2I set for which small-molecule inhibitors have been designed are overlaid as black circles and labelled. Interfaces for which an enclosed residue cluster was found are marked by coloured circles.

To explore how residues utilising concavity may be exploited for drug discovery, enclosed residues at PPI interfaces were clustered in 3D. These enclosed residue clusters represent pockets, or adjacent sub-pockets, that are demonstrably utilized by proteins at interfaces and thus have potential for orthosteric challenge with small-molecules. This revealed that 9253 interfaces possessed enclosed residue clusters (16% of the dataset) (Figure 5). Protein–Peptide interfaces had the smallest proportion of interfaces with enclosed residue clusters (11%), followed by identical pairs with non-symmetric interfaces (12%), non-identical pairs (12%), enzyme-peptides (13%) and identical pairs with symmetric interfaces with the highest proportion (26%).

The existence of small, buried protein-occupied pockets in larger, multi-segment interfaces, consisting of clusters of multiple small-volume pockets may present opportunities for single residue sites to be competed for with fragments, which could be elaborated into interface competitive small molecules for transient interfaces where interface on/off kinetics could allow competitive inhibition. Geometric clustering of deeply bound and solvent inaccessible residues at interfaces revealed cases in the dataset that presented these dense clusters of enclosed residues, which were potentially occupying druggable pockets. However, the presence of such clusters is not an essential requisite for druggability, as evidenced by only one drugged PPI from the 2P2I dataset [13] possessing an enclosed cluster.

## Discussion

In this study we explored the nature of PPI binding interfaces with respect to binding-mode geometry, interatomic interactions and structural and energetic importance of interface residues. While often considered flat and featureless, we showed that while the majority of interfaces extracted from the PDB were indeed flat on average, many interfaces did utilize concavity at their deepest point, suggesting that an element of concavity is important for many PPIs. Peptidic interfaces and those utilising continuous binding regions at the interface made greater use of concavity on average, suggesting that these binding sites may be better defined with respect to potential exploitation in drug discovery. Depth may provide a way of improving encapsulation of a residue in smaller interfaces, as evidenced by the greater proportion of peptide interface core residues in protein–peptide interfaces using deeper binding modes, and making proportionally higher use of the local binding site space (complemented pockets) available in comparison to other residue environments. Our findings support the anchor hypothesis of many interfaces having deeply bound and solvent inaccessible residues, which can be an important venue in drug discovery. We show that many interfaces provide concavity on both sides of the interface to support interactions.

Surprisingly, no significant correlation was observed between deeply bound or solvent inaccessible residues and their energetic contribution to the interaction, despite hotspot residues being significantly more present at the interface core. On the other hand, significantly larger and multi-segmented interfaces have shown fewer number of hotspots per 100 Å [2], suggesting that hotspots are more spread across the interface to aid the formation and stabilization of interactions between larger molecules, which consequently make them more difficult to target for the development of new small molecule drugs.

We hypothesize that differences in interatomic contact usage by smaller, continuous interfaces compared with larger multi-segmented interfaces may reflect differences in the nature of their recognition. As single segments tended to bind using more grooves than multi-segmented PPIs, the significantly greater use of more specifically directional interactions, such as hydrogen bonding, by single segment interfaces may indicate an evolved imperative for the use of directional interactions to lock a segment into a deep binding site without requiring rearrangement of the globular binding partner. Conversely, for larger and multi-segment interfaces, ionic interactions that may be involved in longer range electrostatic steering may contribute more to recognition where overall concavity is not present, and residues occupying concavities are less prevalent.

By analyzing a large-scale dataset of structurally characterized PPIs from the PDB, we found that interfaces forming a continuous binding segment make greater overall use of protrusion into partner protein concavities on average than do globular discontinuous interactions. Deeply bound residues existed in a large proportion of all interactions and there was a relationship between depth and solvent accessibility depending on the continuity of the interface. Over 80% of interfaces utilized at least one deeply bound, solvent inaccessible residue, and over 16% of interfaces made use of multiple, small-volume sub-pockets of the kind bound by previously developed orthosteric PPI inhibitors.

We propose that while continuous binding sites that make use of concave binding modes overall may be more immediately tractable from a druggability perspective, there may be benefit in targeting globular protein interfaces with discrete, complemented sub-pockets, into which residue-sized small-molecule fragments could protrude. Through analyzing the chemistry of interfaces as an aggregate property, summarizing pairwise atomic interactions, we uncovered different chemical preferences between continuous and discontinuous binding sites, suggesting that single continuous segments require more specific directional interactions, whereas discontinuous interfaces burying larger surface areas rely more on aromatic sealing of the interface, and on electrostatic interactions. These discontinuous interfaces may be more amenable to target by allosteric or interface approaches. Our results move towards a better understanding of the features used at therapeutically relevant PPI interfaces, which can then be used on a more rational approach to drug design.

Finally, recent advances in protein structure prediction by AlphaFold [14] and RosettaFold [15] allowed for a drastic increase in the number of protein structures available for many organisms, including *Homo sapiens* with reportedly 98% structural coverage of the proteome currently available in the AlphaFold database [16]. More recently, DeepMind has extended its predictive model to extract evolutionary properties from Multiple Sequence Alignments(MSA), and developed AlphaFold-Multimer [17], allowing for the prediction of homomeric and heteromeric PPIs. However, despite representing an invaluable contribution to the field of structural biology and an improvement in performance when compared with previous methods, AlphaFold-Multimer shows generally higher performance for homomeric interfaces than for heteromeric PPIs, which is likely related to its reliance on MSA for encoding evolutionary information. Moreover, as discussed in the original study, prediction of binding of antibodies is an area of improvement for future implementations of this method. Capturing different conformations remains a major challenge for computational prediction of protein structures, which is particularly important in the context of understanding the molecular mechanisms and biological processes involving PPIs. As these novel artificial intelligence methods mature and address some of their main limitations, analysis such as the ones carried out on this study would greatly expand our understanding of how proteins interact at a molecular level, and could provide valuable biological insights for more complex PPIs, such as the relationship between predicted intrinsically disordered regions and interactions with other proteins.

## Materials and methods

### Data

Pairwise structures of interacting proteins were extracted from the PDB (accessed on 14 April 2021). Interactions with missing atoms at the interface, interfaces that overlapped with other interfaces (overlapping interfaces, where more than two protomers were bound together using the same residues, interfered with interpretation of concavity), interfaces where the product of the number of residues contributed by each protein partner was less than 25 and interfaces where less than 100 Å [2] was buried between the two proteins, were removed from the dataset. The latter two filters were used to remove interfaces where the chains did not make substantial contact [18]. To simplify large scale analysis, only the first model of NMR derived structures was considered.

A non-redundant set of PPI interfaces was generated by clustering interfaces first on whether the interacting pair of proteins was identical using CD-HIT at 95% identity cutoff [19] and subsequently by clustering interactions involving identical protein chains based on the interface sequence. Here, we used the SequenceMatcher module, available in the *difflib* Python package, to compare short peptide sequences, with a similarity cutoff of 75%. Representative interface pairs for each cluster were chosen based on a structure quality score [18].

The final dataset of interfaces was partitioned by categorizing interactions between globular proteins and protein–peptide interactions. The dataset consisted of 55 189 interfaces, of which 15 920 were identical pairs with symmetric interface, 8580 were identical pairs with non-symmetric interface, 28 165 were non-identical pairs, 1702 were protein–peptide interfaces and 822 were enzyme–peptide interfaces (Figure S23, see Supplementary Data available online at https://academic.oup.com/bib). Interactions between peptides and enzymes were separated from interactions with non-enzymatic proteins by identifying enzyme chains using the SIFTS cross-database mappings of the PDB to EC enzyme classification database [20], to differentiate enzyme–substrate and enzyme–inhibitor interactions that may involve active site cavities from non-catalytic site protein–peptide interfaces.

### Interface properties

Pairwise PPI interfaces consist of two interacting protein surfaces. Some properties of these interfaces, such as buried surface area, are property of the whole interface. However, other properties including binding depth belong to one side of the interactions. For the latter, we conducted the analysis from the perspective of the smaller side of the interface (the side contributing the fewest residues; for example, the peptide in a protein–peptide interface), unless otherwise stated. Properties analyzed included shape complementary, interface packing and planarity for whole interfaces. The shape correlation (Sc) measure uses interface region surface normal vectors to determine how well fit is the interface between two proteins [21]. However, in this work, we used a more recent implementation which uses Delauney triangulation to calculate a Normalized Sc (NSc) and Interface Packing (NIP) [22]. Planarity of the interface was measured by using RMSD of interface residues Cα atoms from a least-squares fitted plane through the interface. The resulting planarity value, measured in angstroms (Å), is lower for more planar interfaces, and higher otherwise.

**Figure 6:**
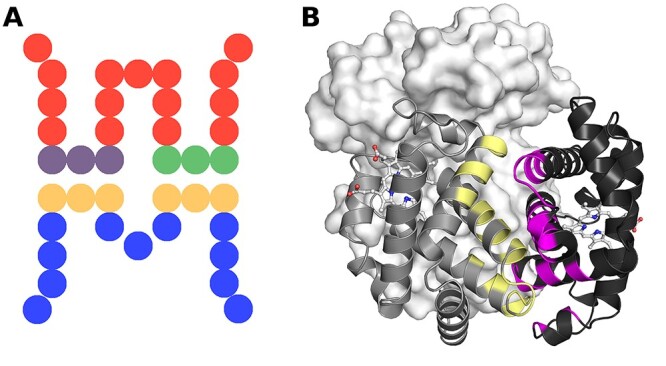
Schematic and structural examples of interface segmentation. Panel (**A**) shows the schematic diagram of a hypothetical pairwise PPI interface. Each circle represents a residue in the primary structure. The interface region of the red chain is split into two segments (purple and green) as it is composed of two discontinuous segments. The interface region of the blue chain is also not continuous if no gap of non-interacting residues is allowed; however, if we allow non-interacting gaps of up to four residues, the interface residues form one continuous segment (yellow). Example of segmentation in a pairwise PPI interface is given in panel (**B**): Chains A and B of a haemoglobin mutant (PDB: 1A01) are shown in cartoon representation coloured in grey and black with segment regions coloured in yellow and magenta. Chains C and D are shown in the background in surface representation. A gap threshold of four was used, and thus, the non-interacting parts of interface helices are not part of each helix’s segment, but interacting residues on the helix are counted as a single interacting segment.

In this work, segmentation refers to the continuity of an interface with respect to primary structure. Segments can optionally have a gap threshold of how far apart two interface residue can be (in the primary sequence) so they are still considered in a single segment (Figure 6). Segment determination was based on the sequence numbering present in the PDB file to determine continuous sections of the primary structure. We used a segmentation definition wherein a segment consists of a section of primary structure at the interaction interface, with gaps of no more than four non-interacting residues allowed within each segment.

As for properties of protein residues, here we calculated the proportion of secondary structure types using DSSP via Biopython [23]. Secondary structure types were categorized into α-helix, β-sheets and loops (disordered regions) as described in Table S56. In addition, solvent accessibility was generated via NACCESS [24], non-covalent interactions were calculated using Arpeggio [25] and concavity was measured using the inaccessible probe radius (*R*_inaccess_) value, in angstroms, calculated using Ghecom [12]. Concavity per residue was measured by using the deepest-bound atom’s concavity value, while whole interface concavity was calculated via arithmetic mean of these deepest per-residue values across all interface residues.

Residues within 5 Å of any of the binding partner’s protein atoms were considered to be part of the interface, and were further categorized as being core or periphery based on their solvent accessibility [18, 26]. Relative Solvent Accessibility (RSA) gives a measurement of burial from solvent that is comparable between residues of different volumes and is used to determine which residues are buried in protein or interface cores. The categories used for residue solvent exposure are outlined in Table S57 (see Supplementary Data available online at https://academic.oup.com/bib).

### Energetically important interface residues

The Ghecom measurement of concavity together with solvent accessibility was used to elucidate potential anchor residues from interface structure. Any residue that was solvent inaccessible with a residue minimum concavity threshold of 4 Å or less was classified as enclosed residues. The DBSCAN density-based clustering algorithm [27] was used to geometrically cluster enclosed residues at interfaces to search for possible orthosteric pockets, defined by clusters of anchors.

Finally, ΔΔG^Binding^ values from mCSM-PPI [28] were used to perform computational alanine scanning of each interface, in order to determine the energetic importance of each binding residue. The threshold of |ΔΔG^Binding^| > 1 kcal/mol was then used to determine whether a residue was a hotspot or non-hotspot [29].

### Statistical analysis

The one-way analysis of variance (ANOVA), as implemented in the stats module of SciPy [30], was used to compare distributions between different groups. Where ANOVA indicated significant differences between groups, we used Tukey’s Honestly Significant Difference (Tukey’s HSD) to categorize observations into their similar or different statistical significance using the Python module statsmodels [31].

**Key Points**

- This review presents a detailed analysis of the landscape of therapeutically relevant PPI interfaces.
- We show that while interfaces forming continuous segments make greater use of concavity, discontinuous interfaces are also amenable to modulation through allosteric or competitive inhibitors.
- We discuss how a better understanding of features used at therapeutically relevant PPI interfaces can then be used on a more rational approach to drug design.

## Supplementary Material

ppi_landscapes_BiB_supps_bbac165

## Data Availability

Data and scripts used to generate the analysis presented in this study are freely available at https://bitbucket.org/ascherslab/ppi-landscape/.

## Notes

**Carlos H. M. Rodruiges** is a postdoctoral fellow at Computational Biology and Clinical Informatics at the Baker Heart and Diabetes and the School of Chemistry and Molecular Biology at The University of Queensland. His research interests are in machine learning, bioinformatics and protein-protein interactions.

**Douglas E. V. Pires** is a Senior Lecturer in Digital Health with the School of Computing and Information Systems at the University of Melbourne and group leader at Bio21 Institute. He is a computer scientist and bioinformatician specialising in machine learning and AI and the development of tools to analyse omics data.

**Tom L. Blundell** is a Professor at the Department of Biochemistry, University of Cambridge. His research focuses on structural biology, bioinformatics and drug discovery for cancer and mycobacterial infections.

**David B. Ascher** is Professor and Deputy Director of Biotechnology at The University of Queensland, and head of Computational Biology and Clinical Informatics at the Baker Heart and Diabetes Institute and head of Systems and Computational Biology at Bio21 Institute. He is interested in developing and applying computational tools to assist leveraging clinical and omics data for drug discovery and personalised medicine.
