# Supplementary material for: Structural landscapes of PPI interfaces
Source: Brief Bioinform. 2022 Jun 2;23(4):bbac165. doi: 10.1093/bib/bbac165 (PMC9294409; doi:10.1093/bib/bbac165)
Supplement: ppi_landscapes_BiB_supps_bbac165 [file ppi_landscapes_bib_supps_bbac165.zip › ppi_landscapes_BiB_supps_bbac165.docx]

Structural Landscapes of PPI Interfaces

Carlos H. M. Rodrigues ^1,2,3^, Douglas E. V. Pires ^1,2,4^, Tom L. Blundell ^5^, David B. Ascher ^1,2,3,5,*^

^1^ Computational Biology and Clinical Informatics, Baker Heart and Diabetes Institute, Melbourne, Victoria

^2^ Systems and Computational Biology, Bio21 Institute, University of Melbourne, Melbourne, Victoria

^3^ School of Chemistry and Molecular Biosciences, Bio21 Institute, University of Queensland, Brisbane, Victoria

^4^ School of Computing and Information Systems, University of Melbourne, Melbourne, Victoria

^5^ Department of Biochemistry, University of Cambridge, Cambridge, UK

^*^To whom correspondence should be addressed D.B.A. Tel: +61 90354794; Email: [david.ascher@unimelb.edu.au](mailto:david.ascher@unimelb.edu.au).

## Figures


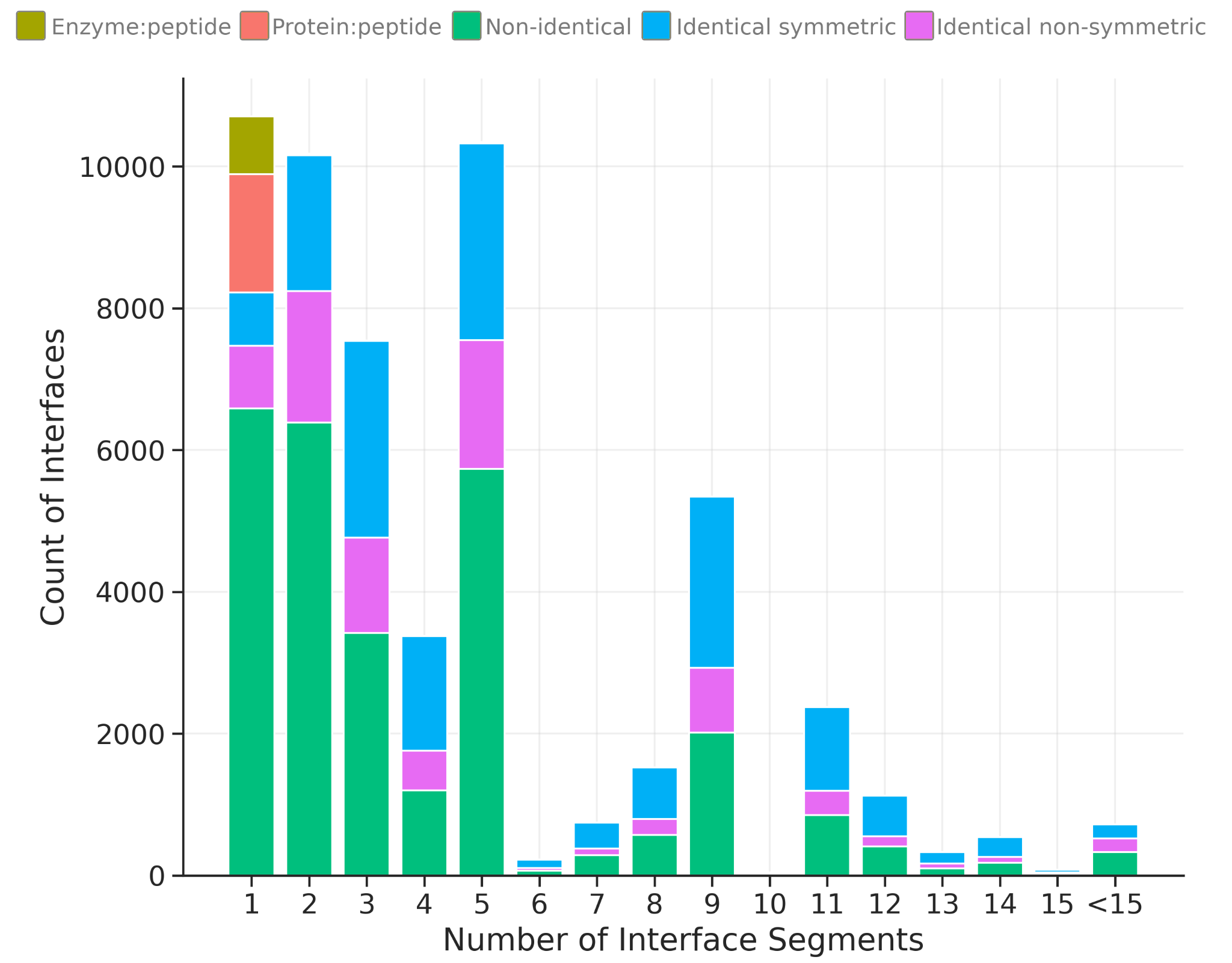


**Figure S1 - Histogram distribution of interface binding continuity for PPI interfaces.** Binding continuity is shown for the smaller side (fewest interacting residues) of each pairwise PPI interface in our non-redundant set. Interfaces are distinguished by interaction type.


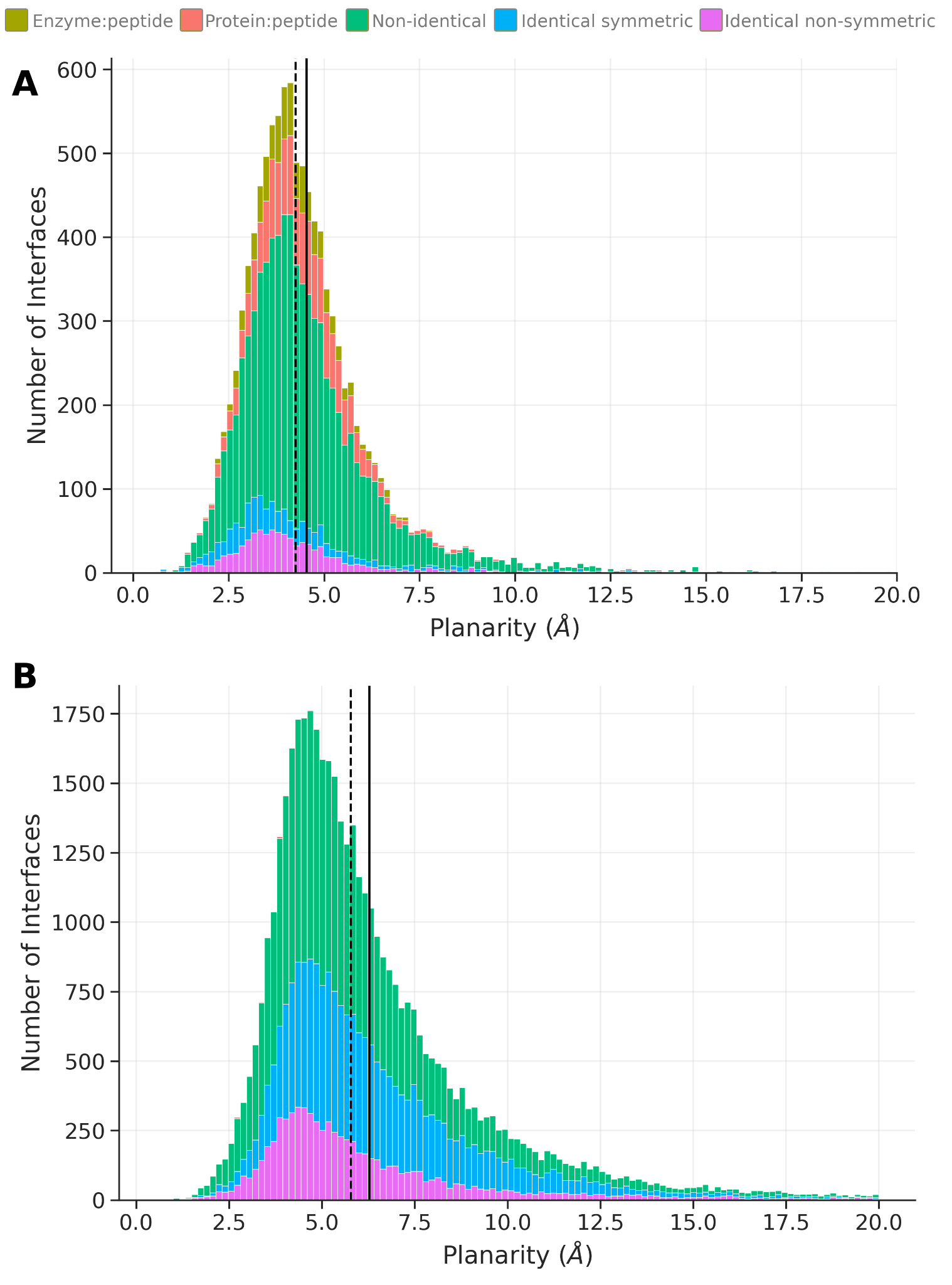


**Figure S2 - Histogram distributions of interface planarity for single and multi segmented interfaces.** Histograms are stacked and filled based on the type of interface. A) displays the distribution of interface planarity for single segmented interfaces and B) for discontinuous interfaces. Solid line indicates distribution mean and dashed line shows the geometrical mean.


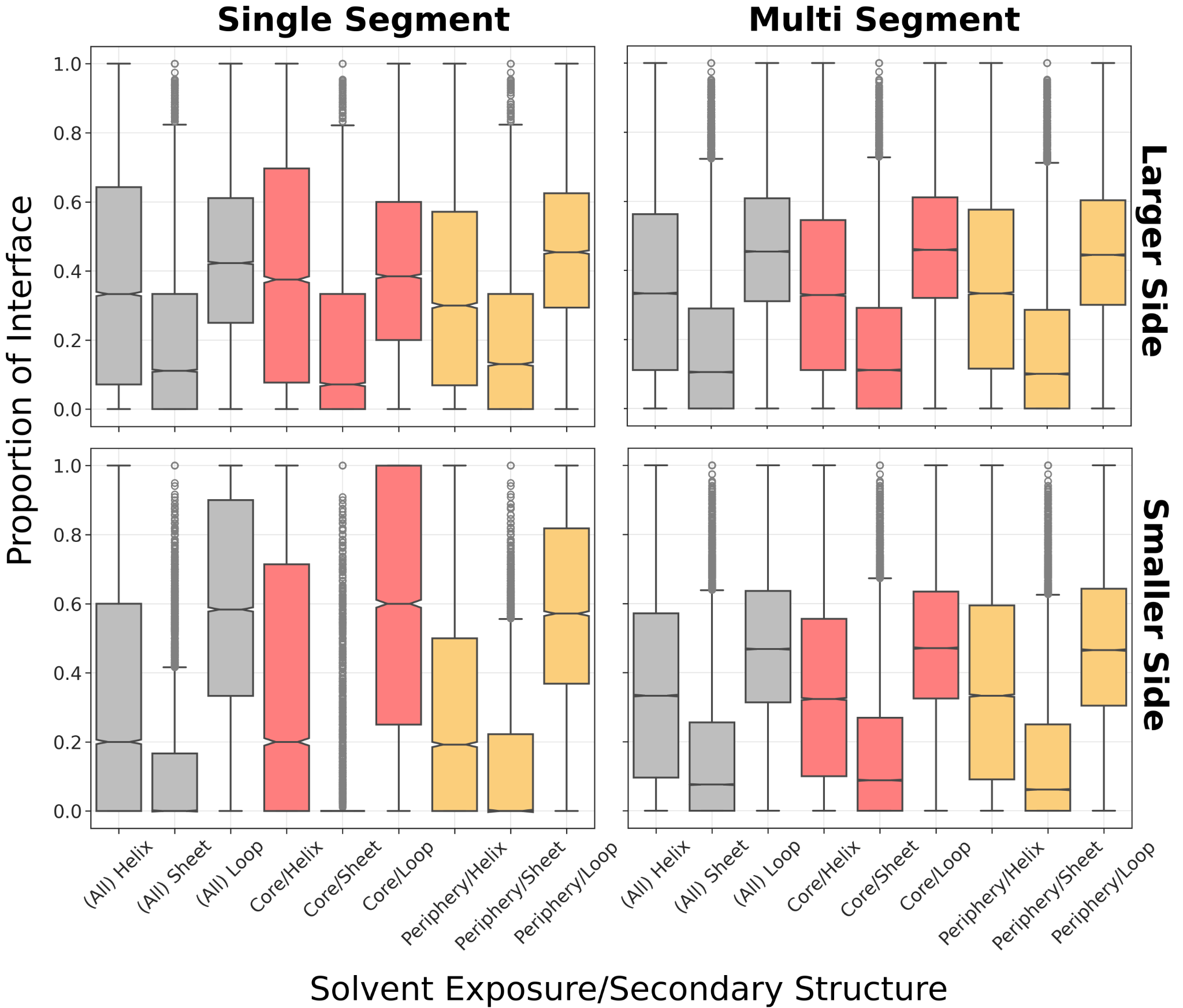


**Figure S3 - Boxplot distributions of interface proportions of secondary structure types, by interface segmentation.** Gray boxes indicate proportions of interfaces with a given secondary structure type. Coloured boxes represent proportions of interfaces with a given secondary structure by interface solvent exposure type.


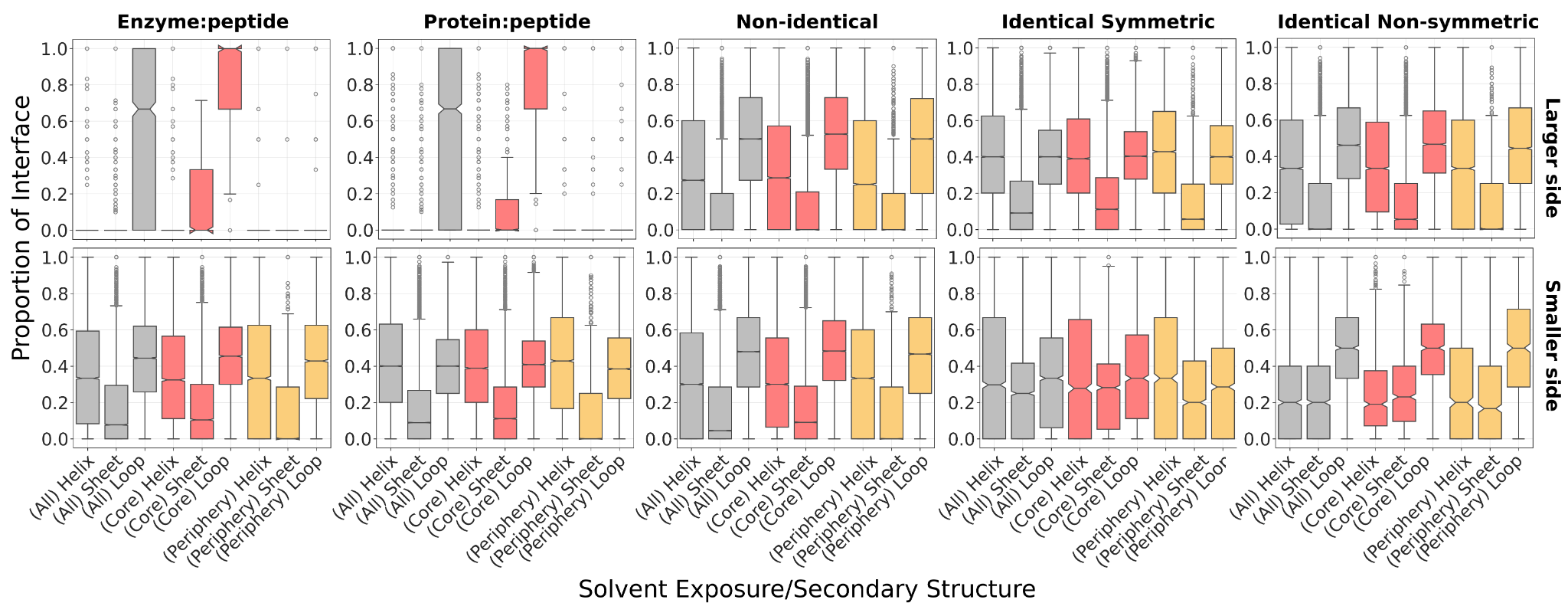


**Figure S4 - Boxplot distributions of interface proportions of secondary structure types, by interface segmentation and interface type.** Gray boxes indicate proportions of interfaces with a given secondary structure type. Coloured boxes represent proportions of interfaces with a given secondary structure by interface solvent exposure type.


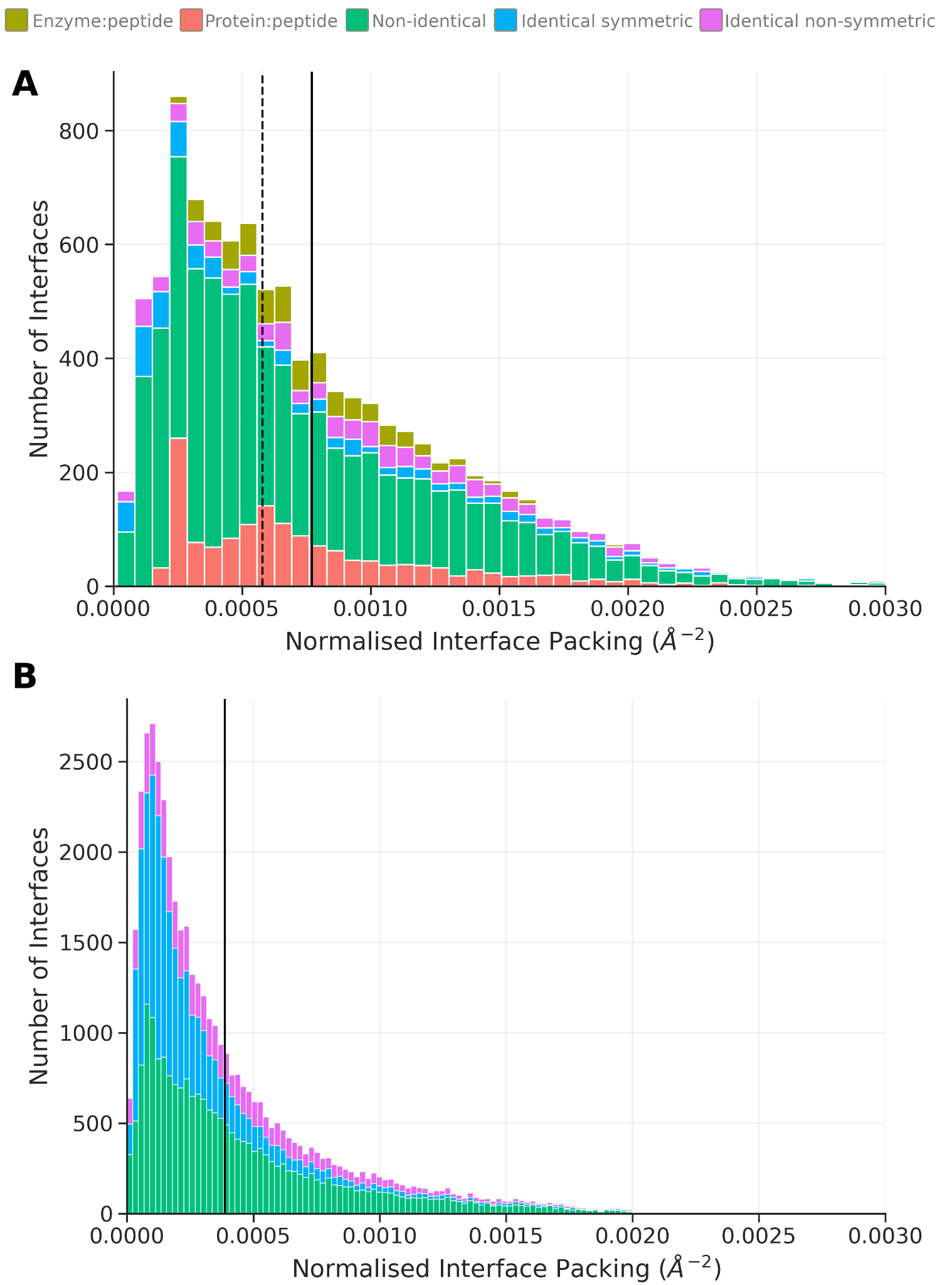


**Figure S5 - Histogram distributions of interface normalised interface packing (NIP) for single and multi segmented interfaces.** Histograms are stacked and filled based on the type of interface. A) displays the distribution of interface packing for single segmented interfaces and B) for discontinuous interfaces. Solid line indicates distribution mean and dashed line shows the geometrical mean.


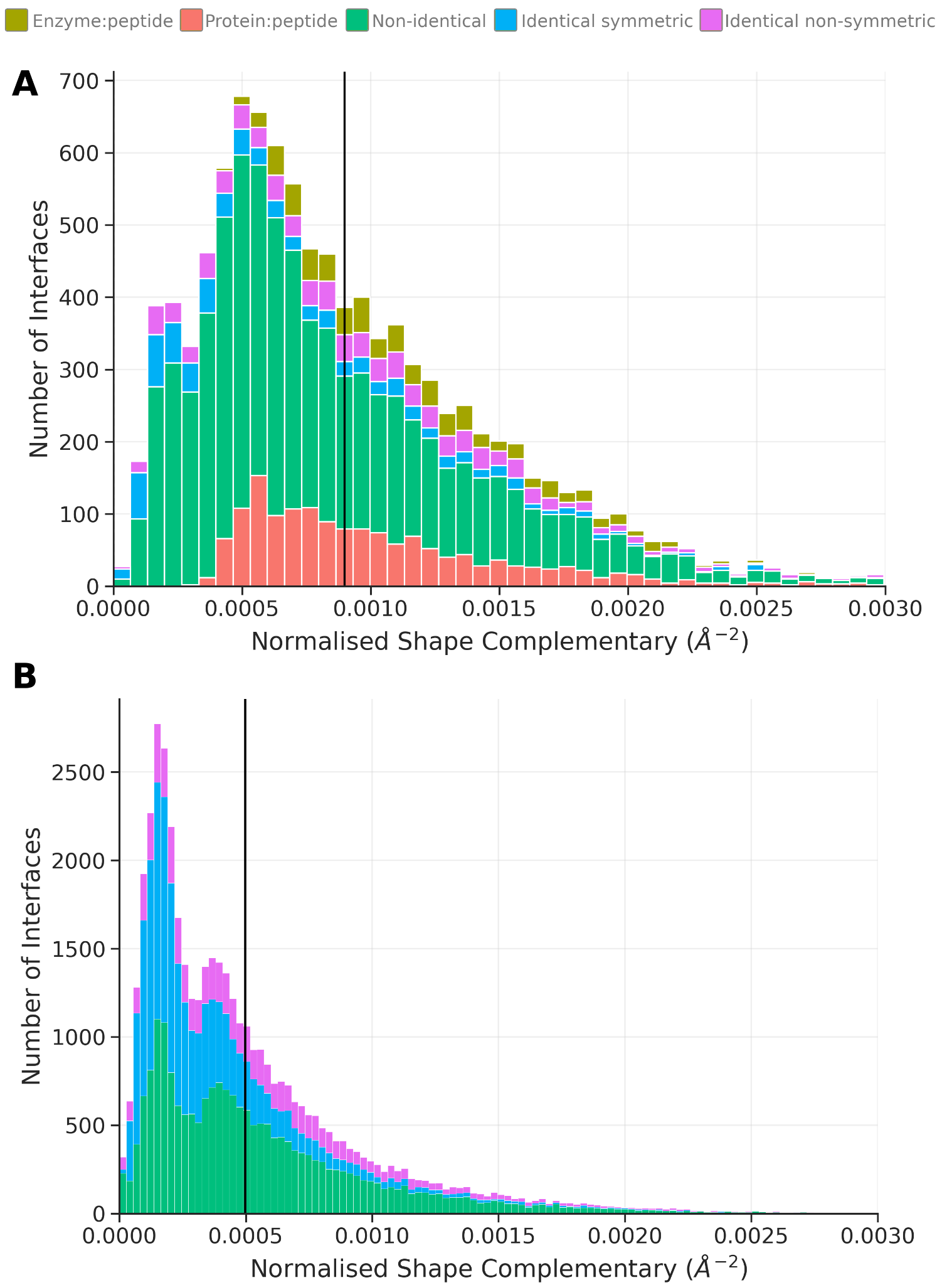


**Figure S6 - Histogram distributions of interface normalised shape correlation (NSc) for single and multi segmented interfaces**. Histograms are stacked and filled based on the type of interface. A) displays the distribution of interface shape complementarity for single segmented interfaces and B) for discontinuous interfaces. Solid line indicates distribution mean and dashed line shows the geometrical mean.


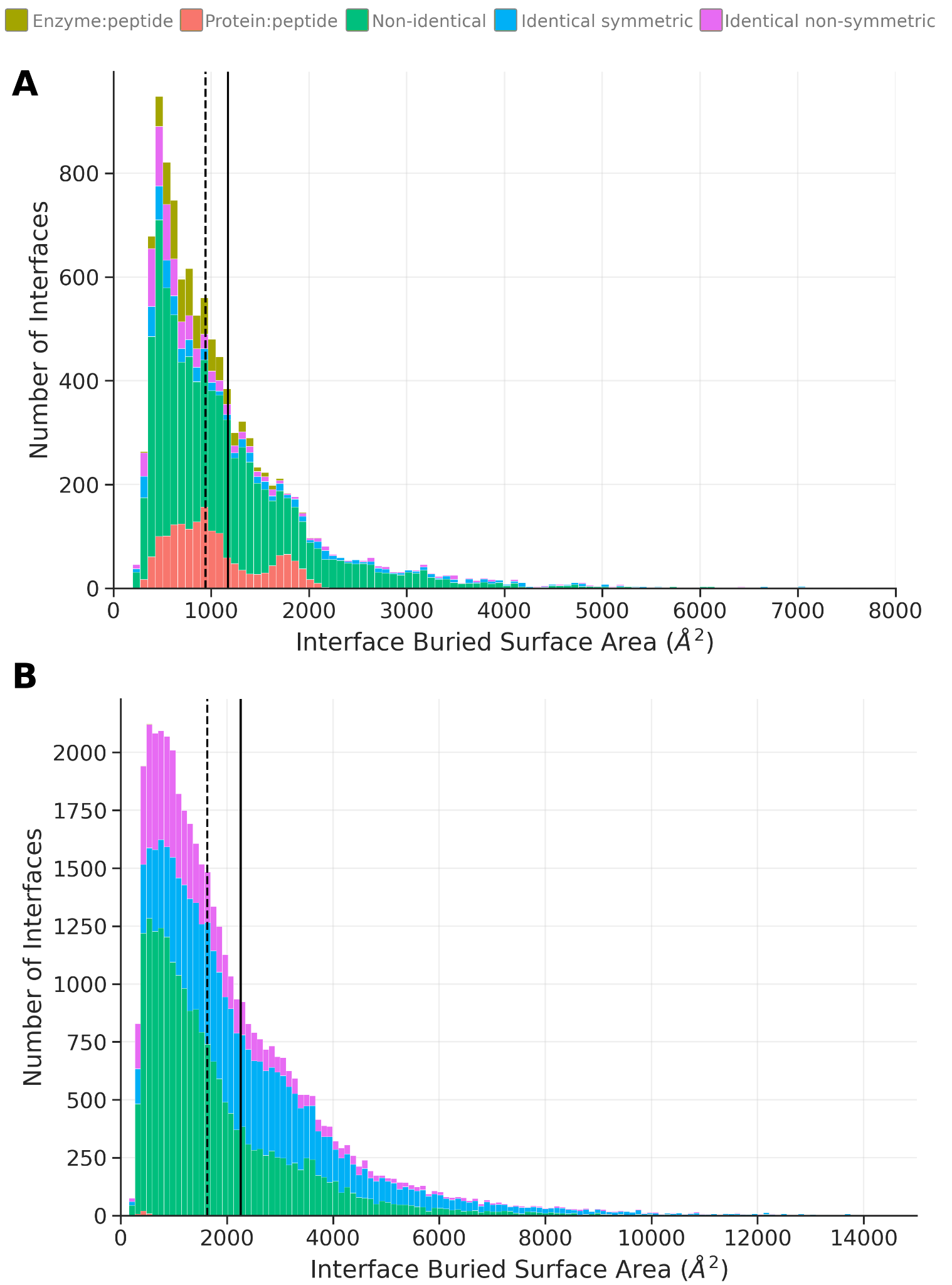


**Figure S7 - Histogram of buried surface area distributions for single and multi segmented interfaces.** Histograms are stacked and filled by type of interface. A) displays the distribution of interface shape complementarity for single segmented interfaces and B) for discontinuous interfaces. Solid line indicates distribution mean and dashed line shows the geometrical mean.


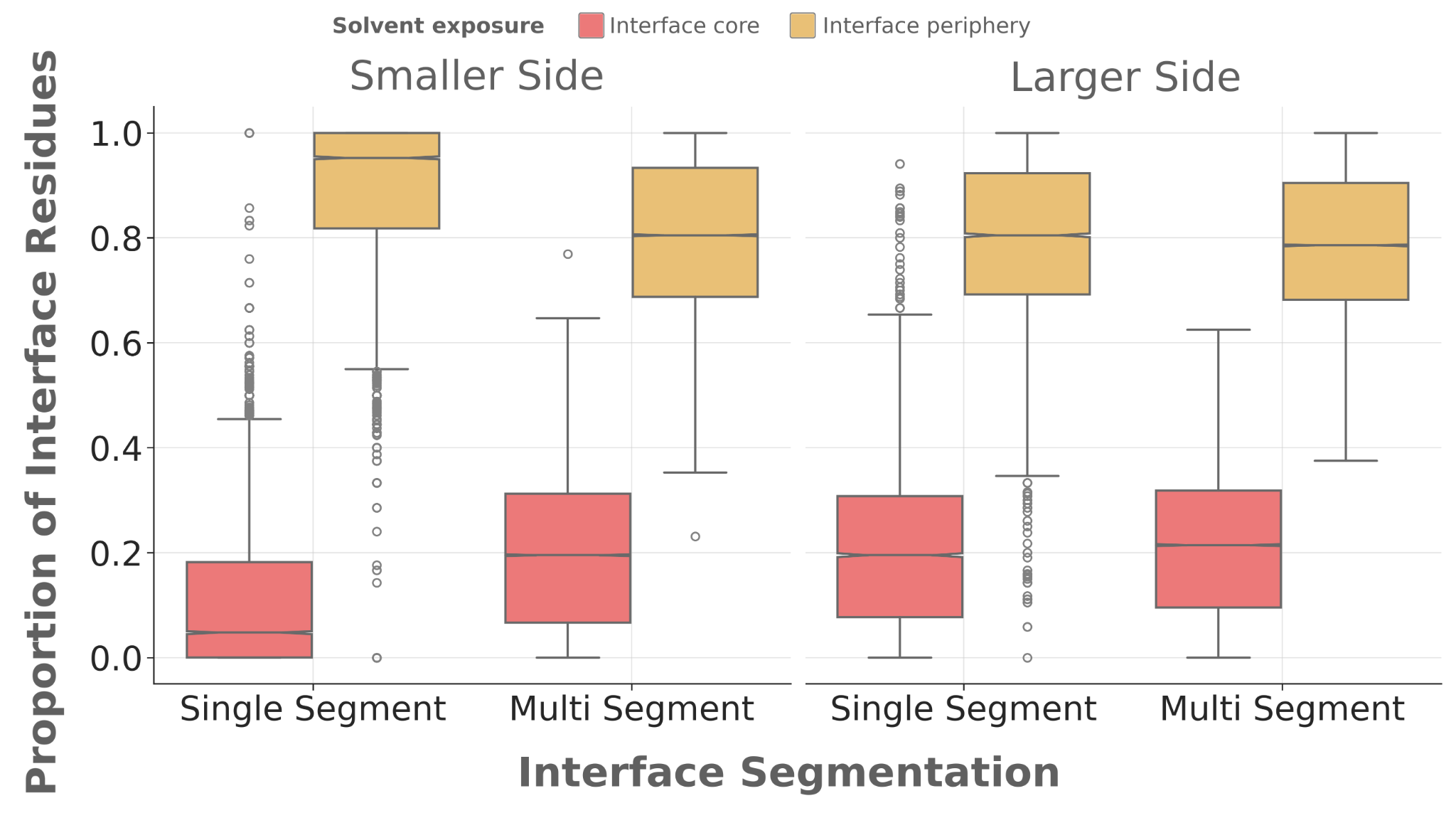


**Figure S8 - Boxplot distributions of interface proportions of residues that were core or periphery, by interface segmentation.** Outliers are shown as translucent gray circles. “Smaller side” refers to the side of the pairwise interface with fewer interacting residues than the “Larger side”.


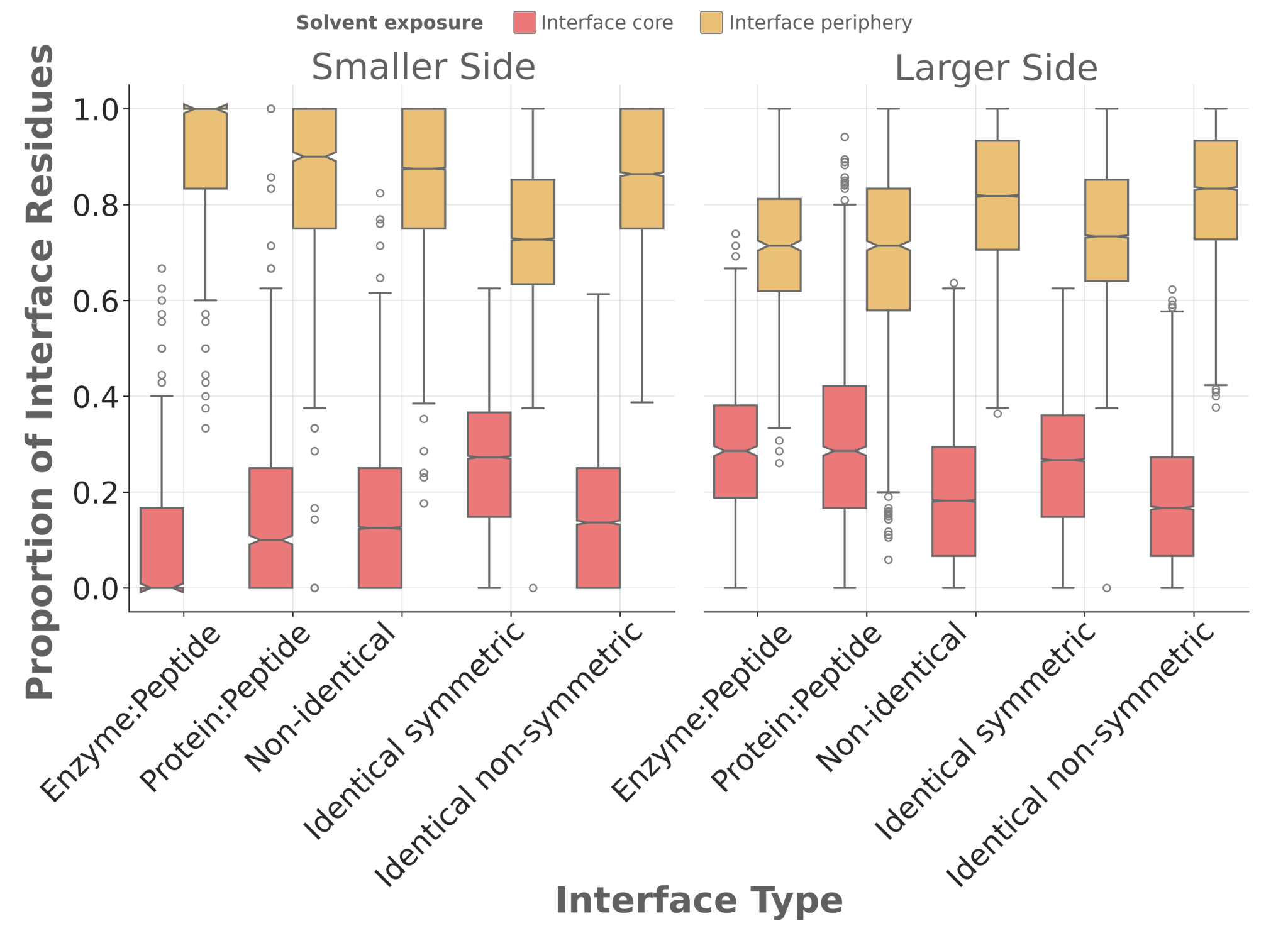


**Figure S9 - Boxplot distributions of interface proportions of residues that were core or periphery, by interface segmentation and interface type.** Outliers are shown as translucent gray circles. “Smaller side” refers to the side of the pairwise interface with fewer interacting residues than the “Larger side”.


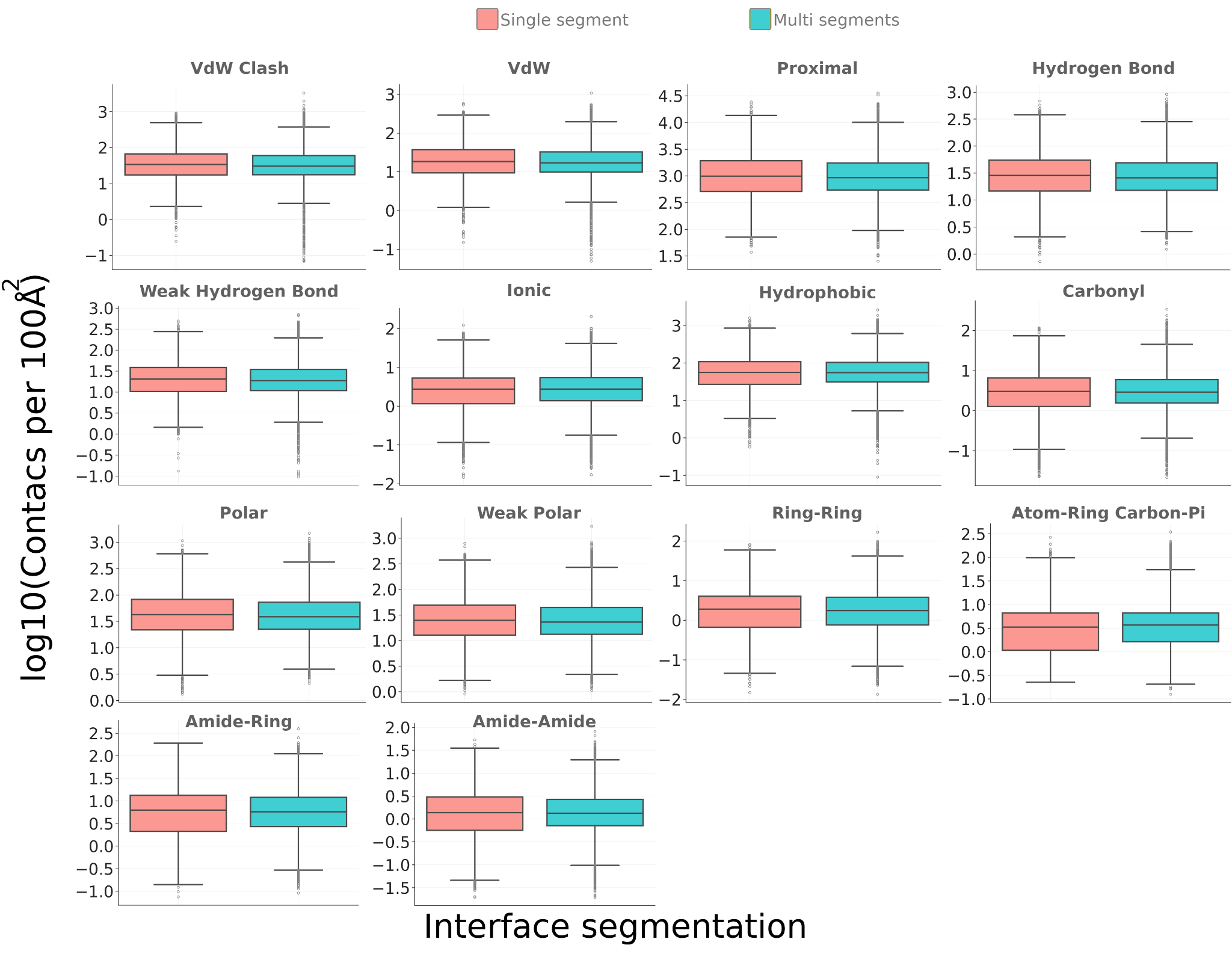


**Figure S10 - Boxplot distributions of Arpeggio structural interactomics analysis of non-covalent interactions per 100Å^2^ of PPI interfaces, comparing interfaces by segmentation.** Outliers are shown as gray circles.


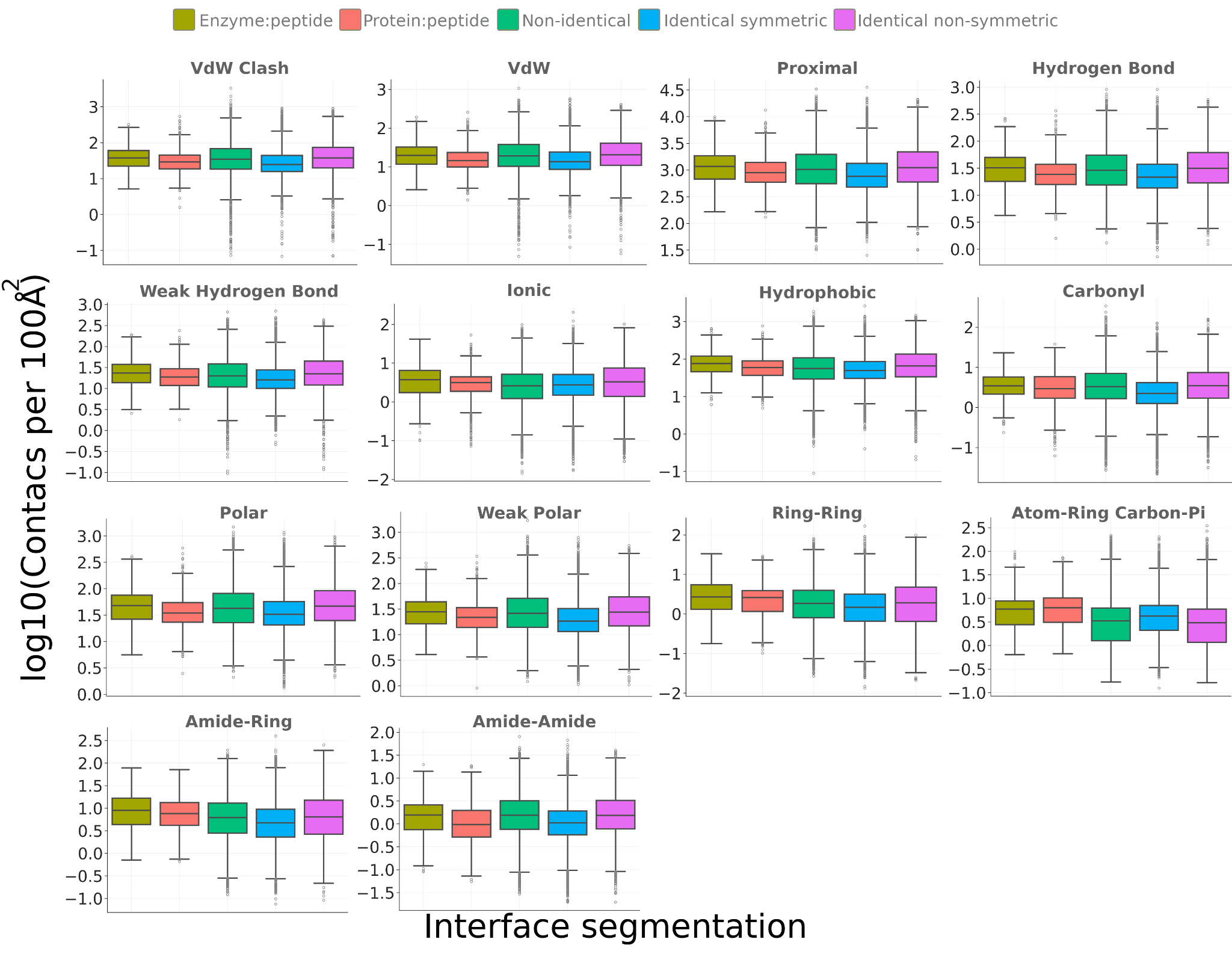


**Figure S11 - Boxplot distributions of Arpeggio structural interatomic analysis of non-covalent interactions per 100Å^2^ of PPI interfaces, comparing interfaces by interface type.** Outliers are shown as gray circles.


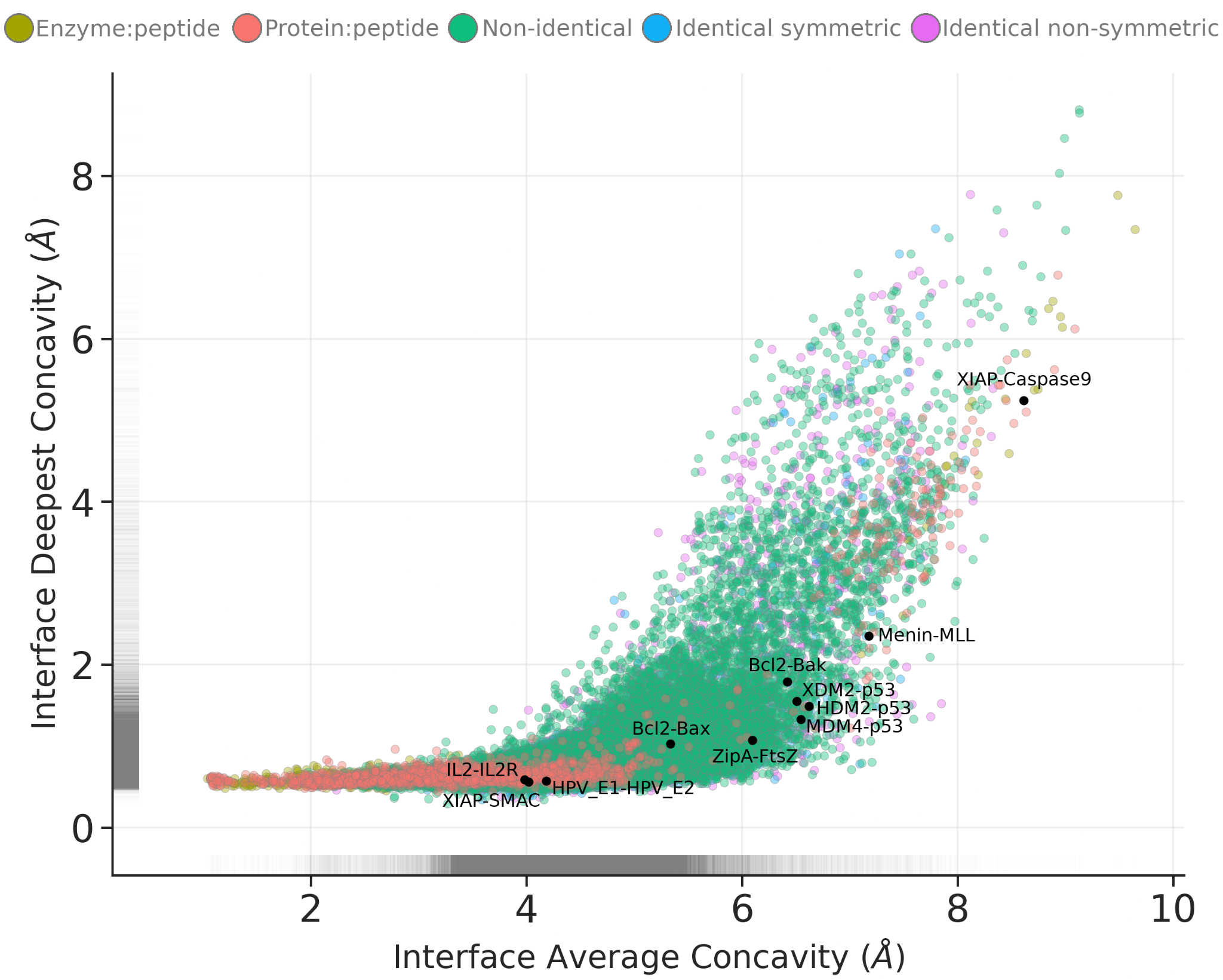


**Figure S12 - Point and 2D density distributions of occupation of concavity at PPI interfaces, on average and at deepest.** Each point represents the smaller side of one interface from the non-redundant set of non-overlapping PPI interfaces. Concavity is measured by Ghecom, representing the smallest spherical probe size that was able to enter a space around the partner protein’s surface 9where smaller values represent deeper binding). Interfaces are coloured by interface type, and PPI interfaces from the 2P2I dataset for which small-molecule inhibitors have been developed are overlaid as black points and are labelled.


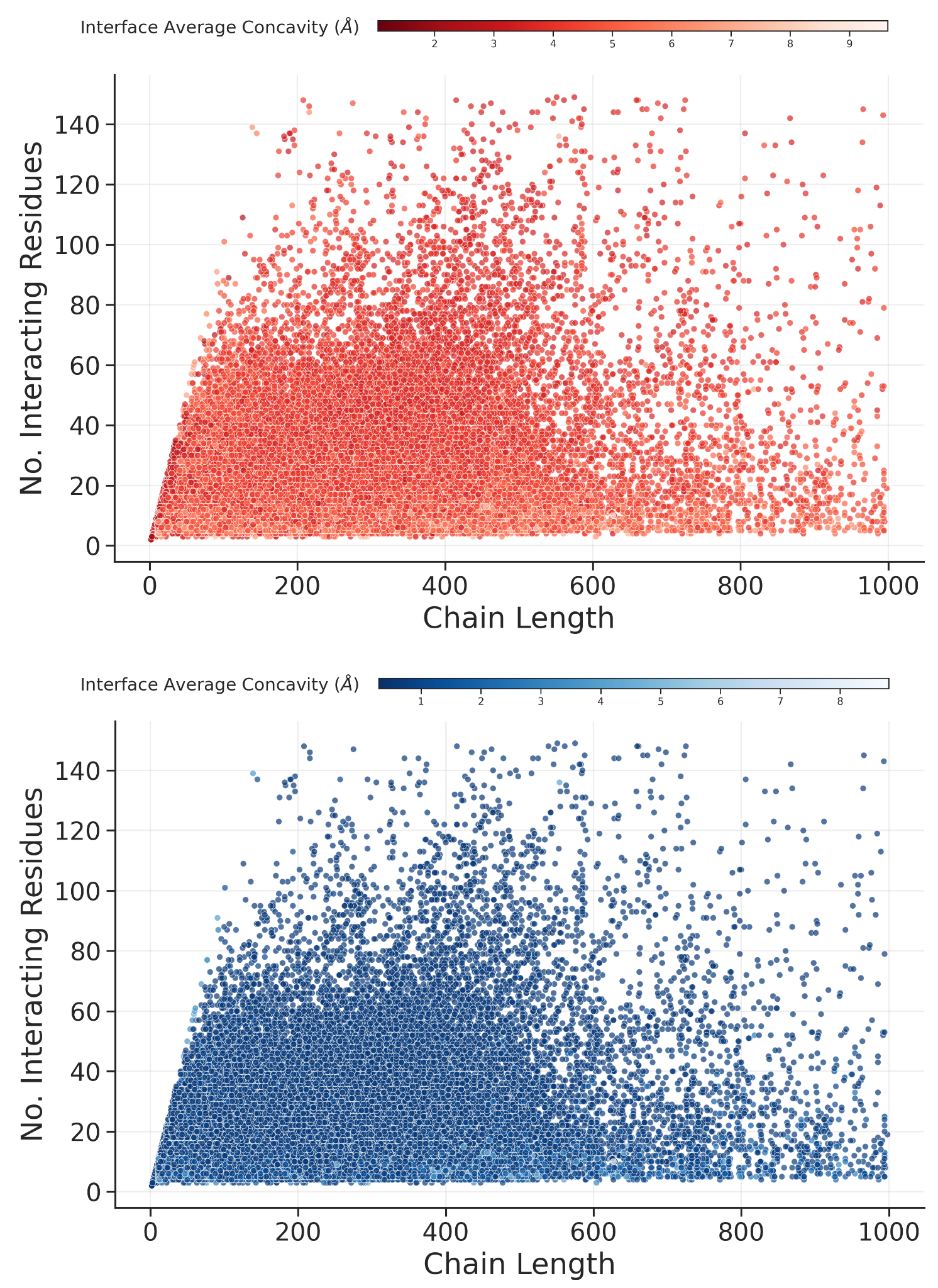


**Figure S13 - Summary plots showing exploitation of concavity by PPI interfaces as the size of interacting protomers and their interacting surface varied.** Each coloured block represents the arithmetic mean of A the average (red) or B the deepest (blue) concavity exploited by interfaces within the plot space covered by the block.


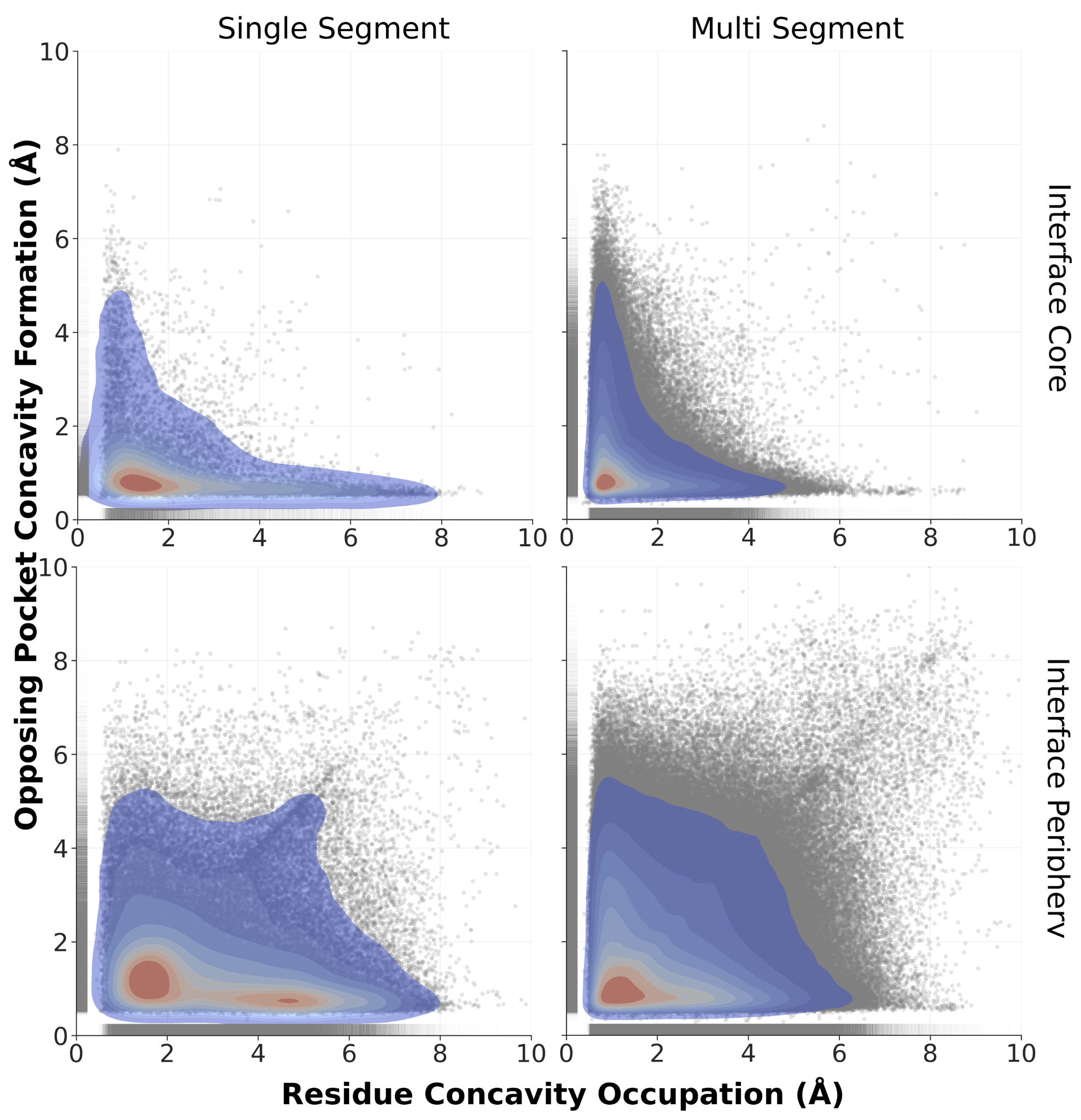


**Figure S14 - Distribution of binding mode and extent of local concavity of residue in different interface segmentations.** The use of concavity interface residues from the shorter chain of interactions of each class is shown on the abscissa. The depth of concavity formation by the deepest surface atom of the partner protein within 5Å of the abscissa residue is shown on the ordinate. Each open circle represents a residue, and the 2D density distribution is shown where red, orange, yellow and blue represent areas of higher through lower point density, respectively.


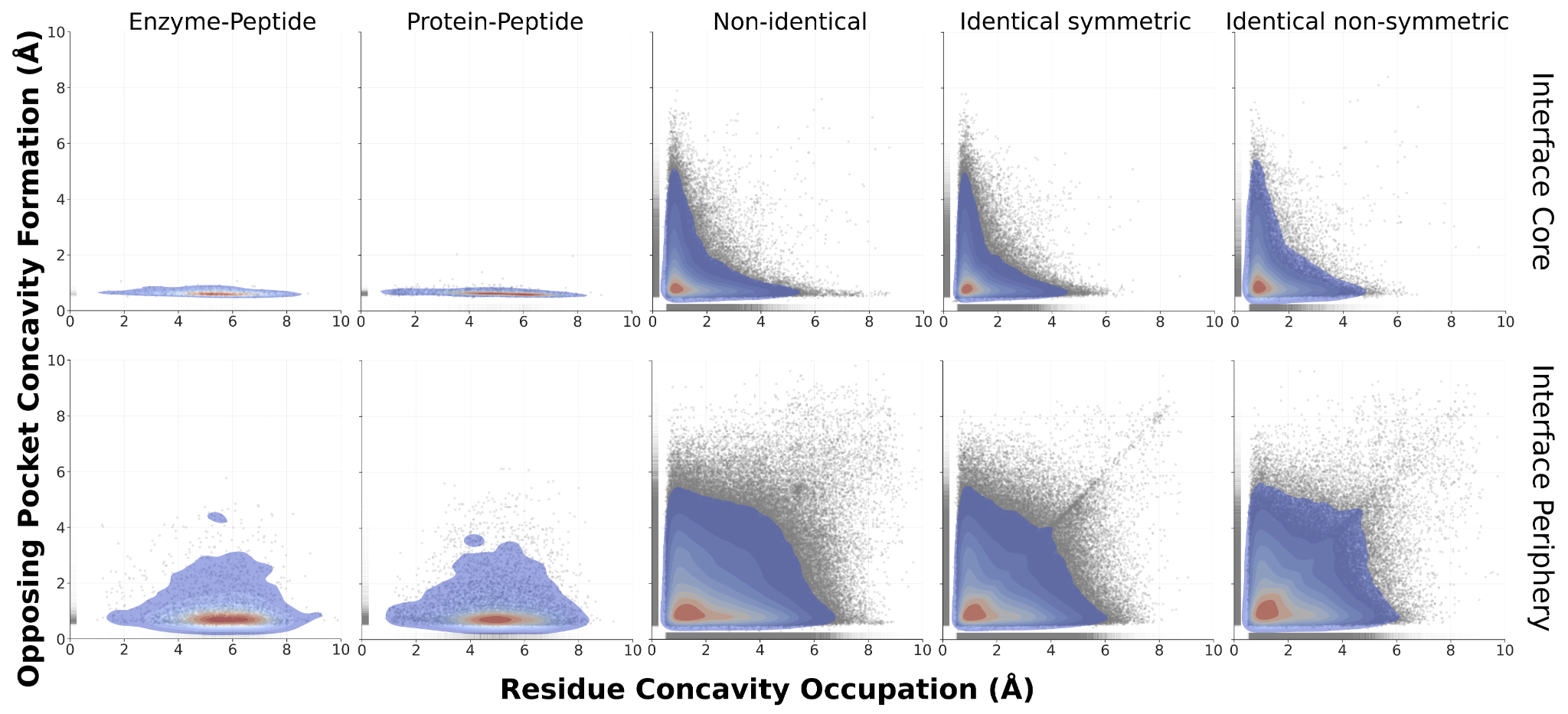


**Figure S15 - Distribution of binding mode and extent of local concavity for residues in different interface classes**. The use of concavity interface residues from the shorter chain of interactions of each class is shown on the abscissa. The extent of concavity formation by the deepest surface atom of the partner protein within 5Å of the abscissa residue is shown on the ordinate. Each open circle represents a residue, and the 2D density distribution is shown where red, orange, yellow and blue represent areas of higher through lower point density, respectively.


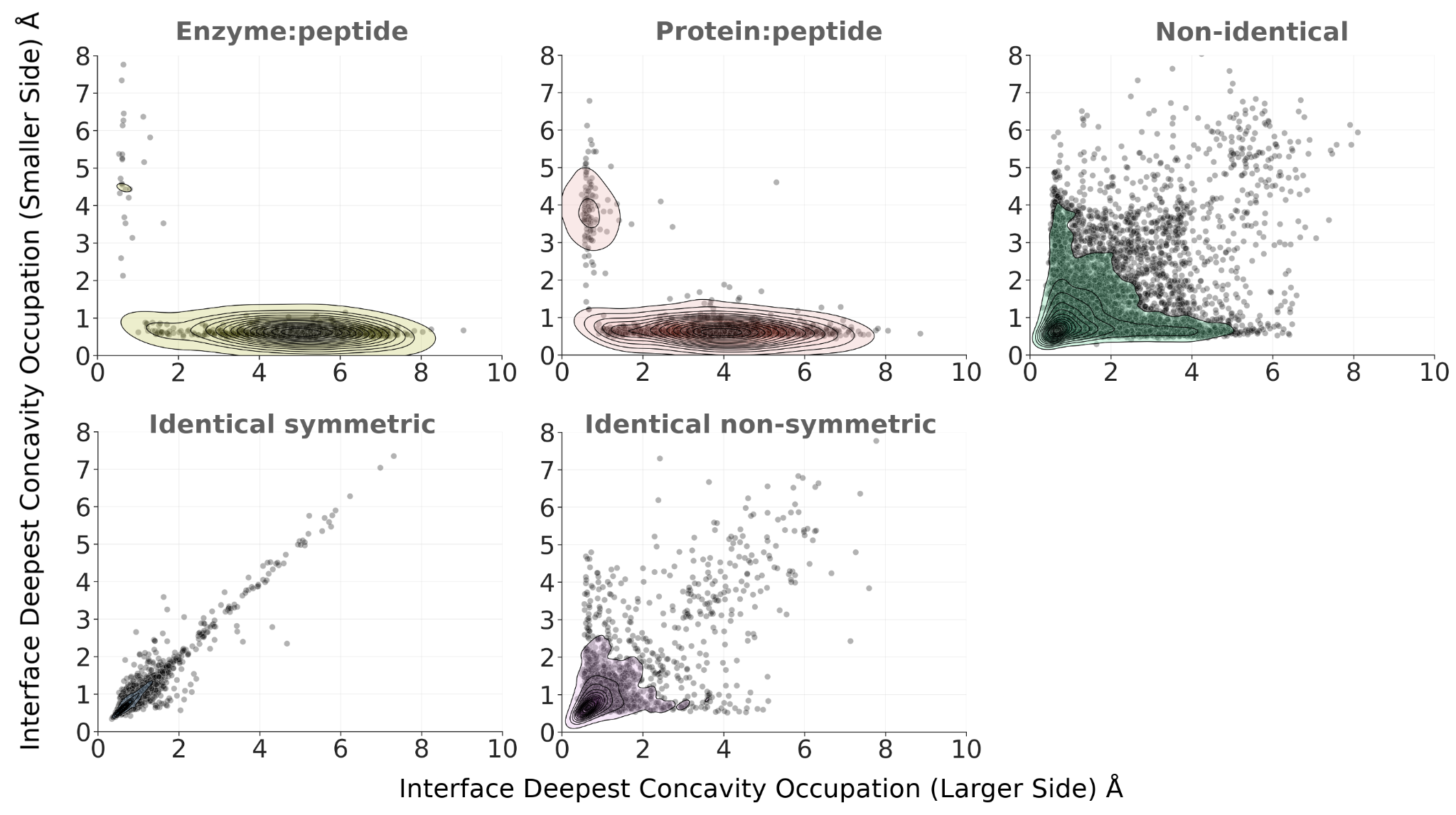


**Figure S16 - Point and 2D density distributions of deepest concavity occupation on the larger and smaller sides of PPI interfaces.** Concavity is measured by Ghecom, representing the smallest spherical probe size that was able to enter a space around the partner protein’s surface (where smaller values represent deeper binding). Density distributions are coloured according to interface type.


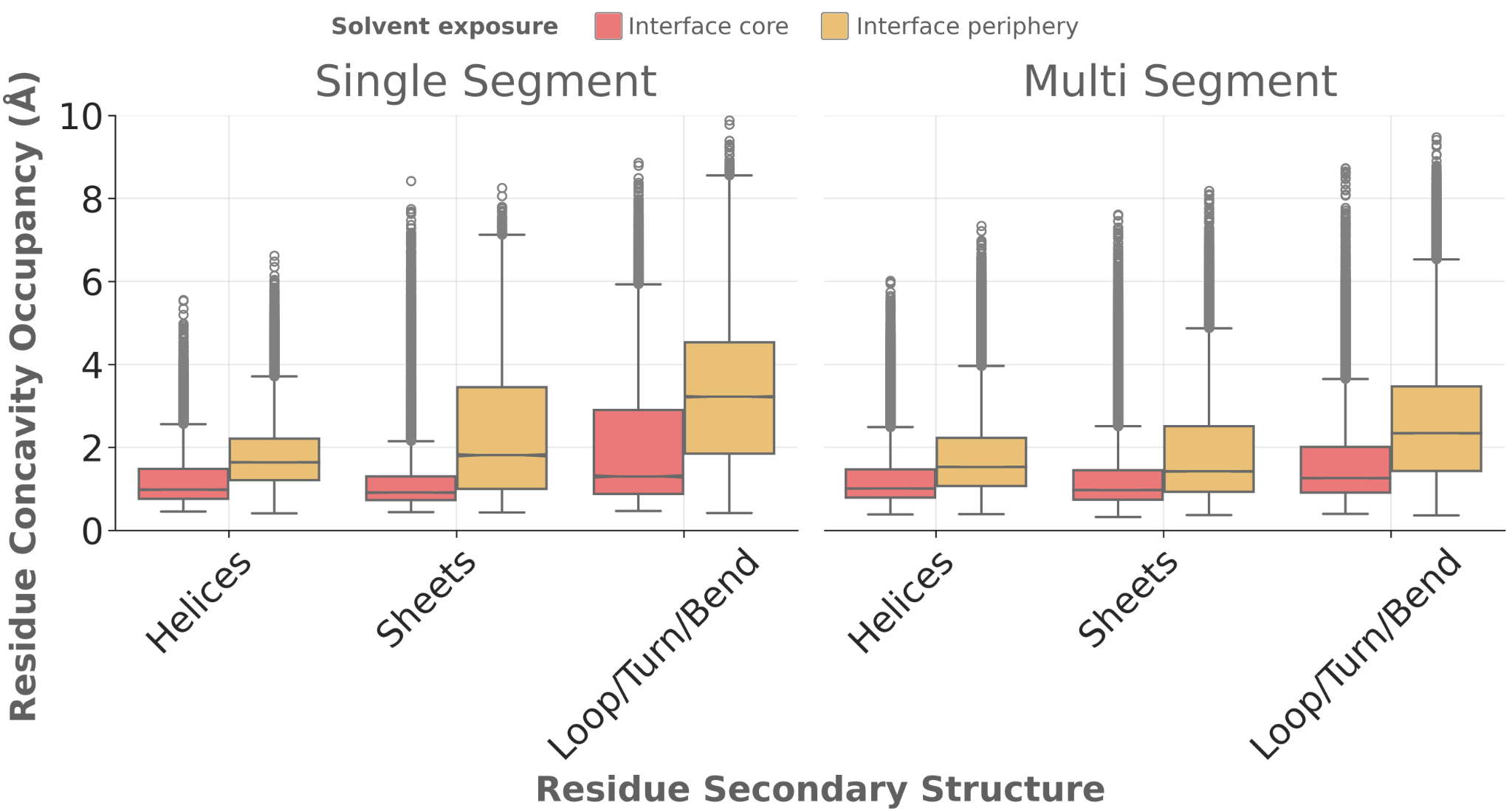


**Figure S17 - Boxplot distributions of interface residue use of concavity by secondary structure, solvent exposure and interface segmentation.** Concavity is measured by Ghecom, representing the smallest spherical probe size that was able to enter a space around the partner protein’s surface (where smaller values represent deeper binding).


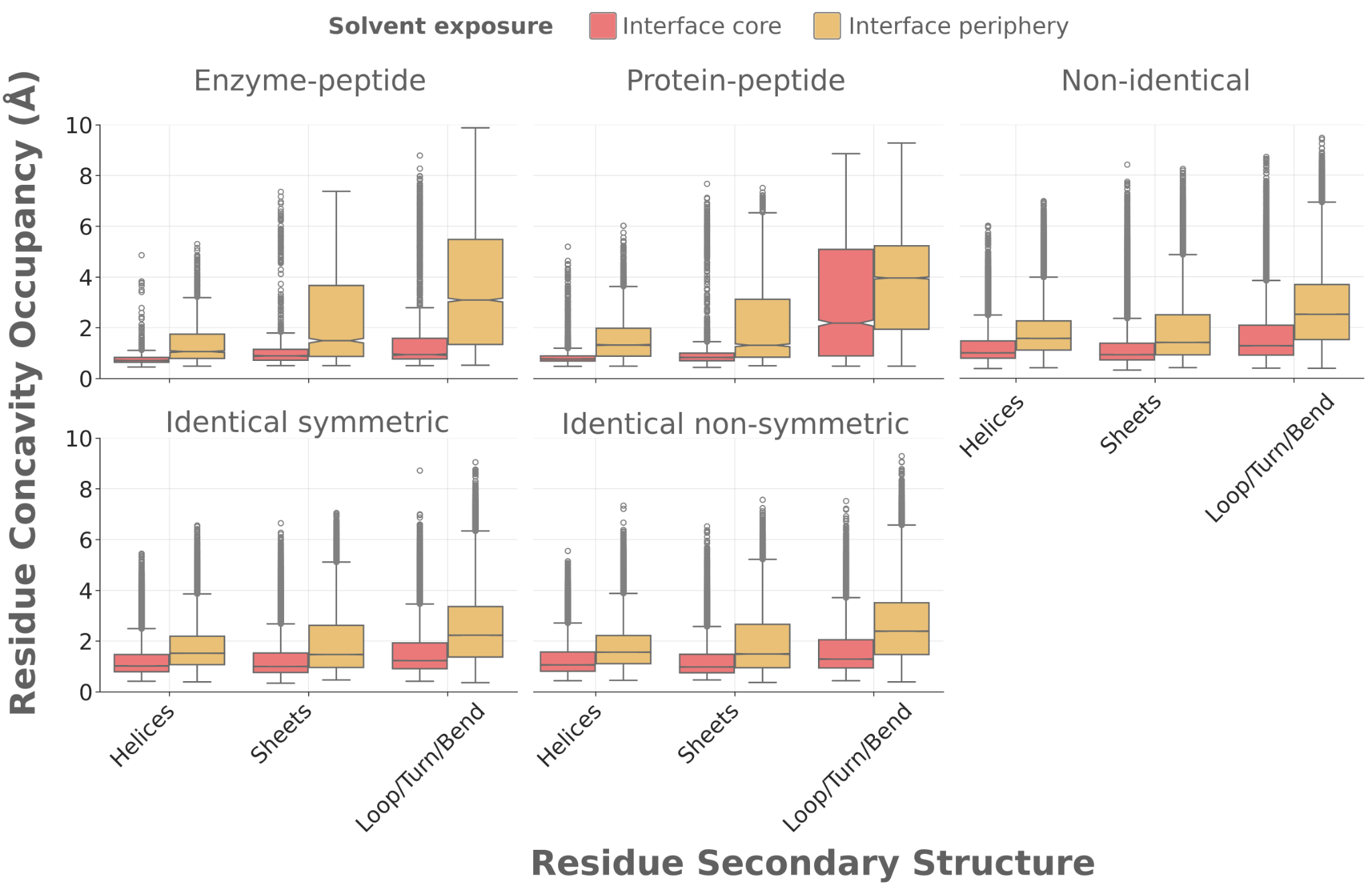


**Figure S18 - Boxplot distributions of interface residue use of concavity by secondary structure, solvent exposure and interface type.** Concavity is measured by Ghecom, representing the smallest spherical probe size that was able to enter a space around the partner protein’s surface (where smaller values represent deeper binding).


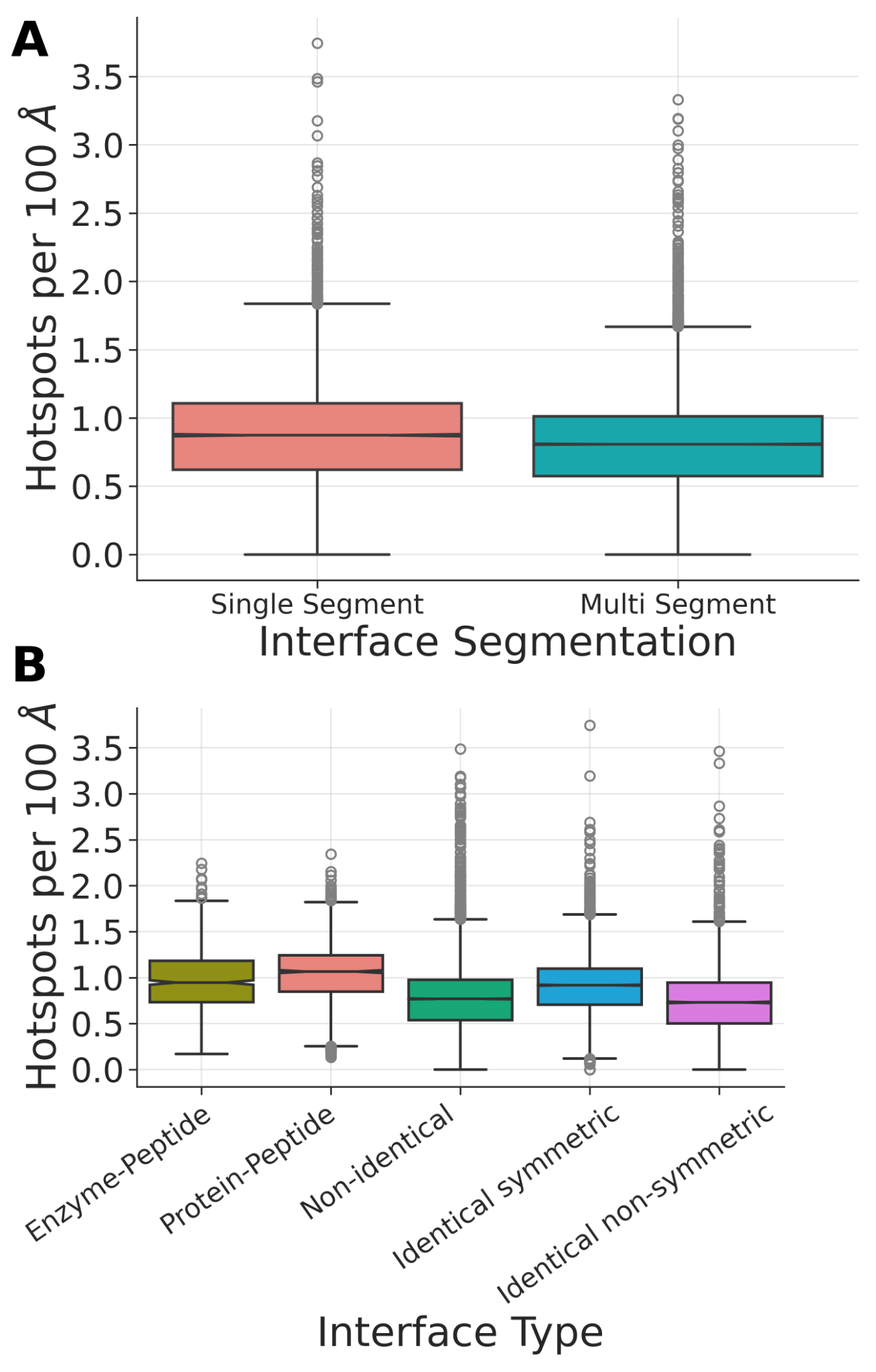


**Figure S19 - Boxplot distributions of hotspots per 100Å^2^ buried surface area.** Distributions of mCSM-PPI predicted hotspots (ΔΔG^Binding^ > 1 kcal/mol) for PPI interfaces by A) interface segmentation and B) interface type.


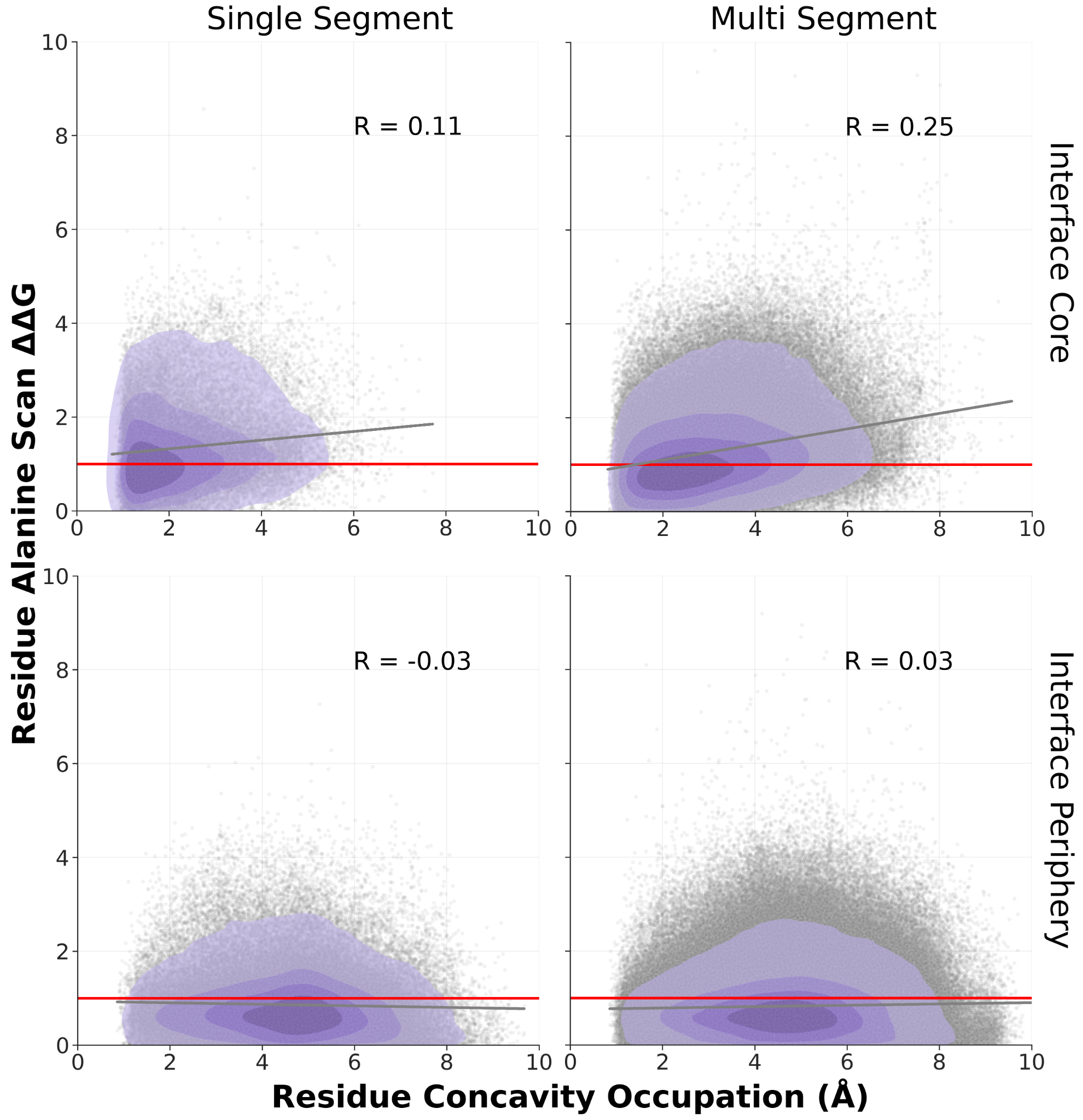


**Figure S20 - Relationships between residue energetic hotspot predictions and use of concavity for interfaces by interface segmentation.** Residue use of concavity as measured by Ghecom is shown on the abscissa, and mCSM-PPI alanine scanning ΔΔG^Binding^ predictions are shown on the ordinate. All residues originate from the deepest bound binding partner. The horizontal red lines show the threshold for mCSM-PPI predictions which consider a residue as a hotspot. Plots are divided by interface type. Linear model fitting is shown by gray lines. R values for Pearson correlation coefficient estimates are shown.


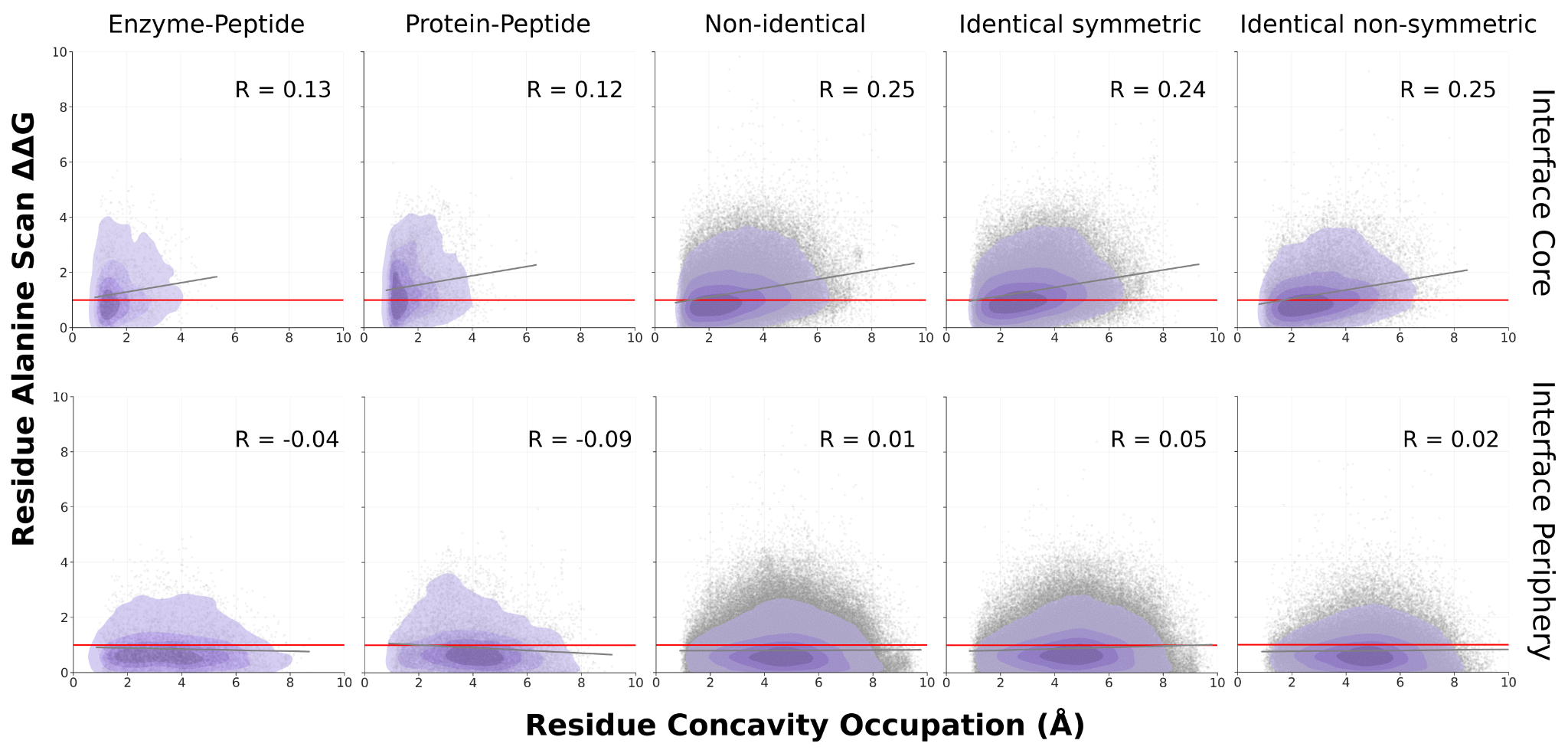


**Figure S21 - Relationships between residue energetic hotspot predictions and use of concavity for different interfaces types**. Residue use of concavity as measured by Ghecom is shown on the abscissa, and mCSM-PPI alanine scanning ΔΔG^Binding^ predictions are shown on the ordinate. All residues originate from the deepest bound binding partner. The horizontal red lines show the threshold for mCSM-PPI predictions to consider a residue as a hotspot. Plots are divided by interface classes. Linear model fitting is shown by gray lines. R values for linear Pearson correlation coefficient estimates are shown.


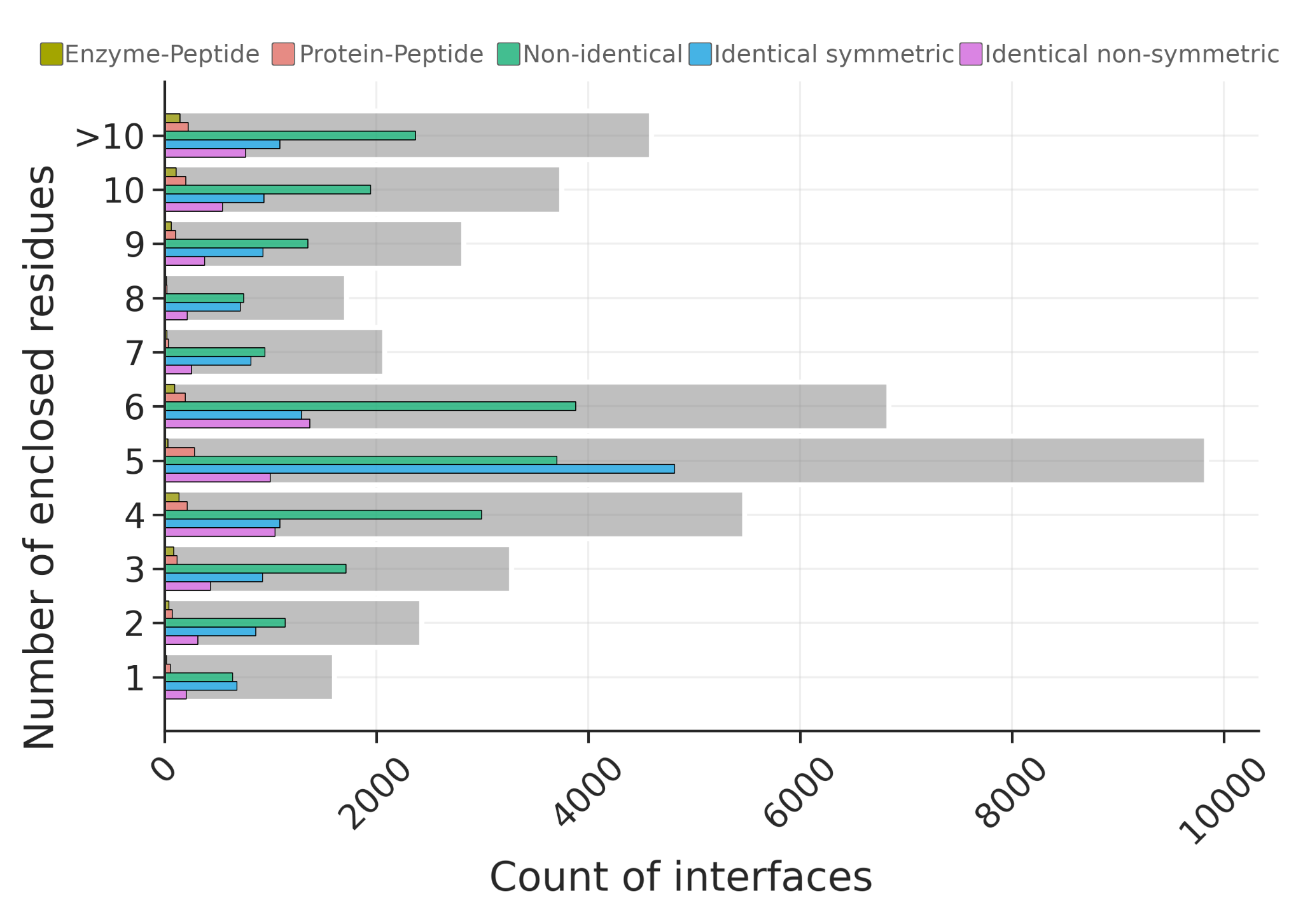


**Figure S22 - Histogram distributions of numbers of deeply bound, solvent inaccessible residues in PPI interfaces.** Distributions of the number of enclosed residues for the non-redundant set of PPI interfaces. Enclosed residues were solvent buried (interface core) residues with a concavity value of ≤ 4Å. The distribution for all interfaces is shown in gray, and the distributions for interface types in colours as per the legend.


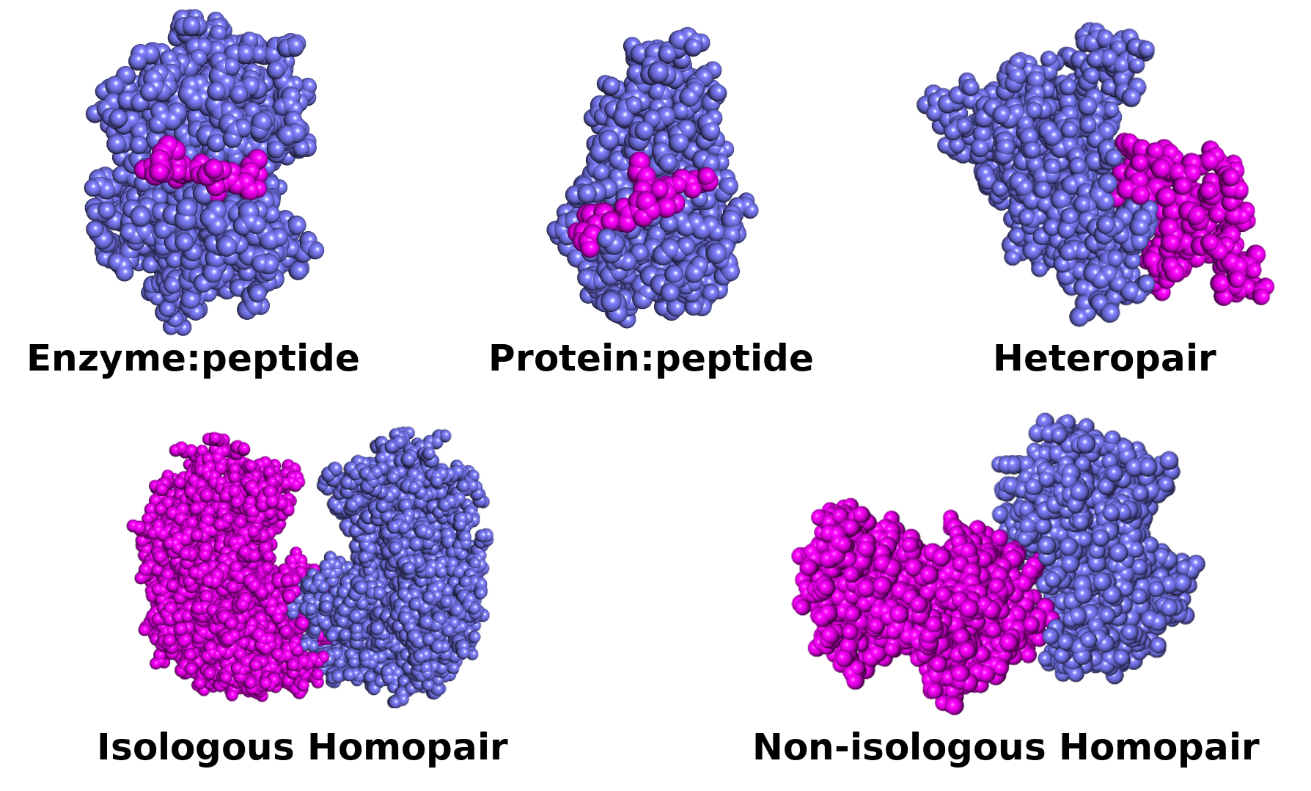


**Figure S23 - Interface types according to structural characterisation of pairwise PPIs.** Isologous homopairs (PDB ID: 3FPC) represent associations of two proteins with the same sequence (or highly identical sequences - 95% identity), using nearly identical residues on each side of the interface. Non-isologous homopairs (PDB ID: 1CZY) comprise interactions between two proteins with high sequence similarity, but using different interface residues. Association of two distinct globular proteins were classified as heteropairs (PDB ID: 1OQD). Interfaces which the interacting pair comprised a globular protein and a peptide (sequence length < 41) were further split into Enzyme:peptide (PDB ID: 8PCH) and Protein:peptide interactions (PDB ID: 1P4U).

## Tables

**Table S1** - Summary statistics and ANOVA analysis of PPI interface numbers of interface segmentation by interface type. ANOVA p-value < 0.05

| Interface type | ANOVA Group | Mean | SD | N | Min | Med | Max | Mode |
| --- | --- | --- | --- | --- | --- | --- | --- | --- |
| Enzyme-peptide | a | 1.01 | 0.13 | 822 | 1 | 1 | 3 | 1 |
| Protein-peptide | a | 1.03 | 0.24 | 1702 | 1 | 1 | 4 | 1 |
| Non-identical | b | 3.41 | 2.99 | 28165 | 1 | 3 | 32 | 1 |
| Identical-symmetric | c | 4.99 | 2.92 | 15920 | 1 | 4 | 33 | 4 |
| Identical-nonsymmetric | d | 4.3 | 3.95 | 8580 | 1 | 3 | 47 | 2 |

**Table S2** - Summary statistics and ANOVA analysis of PPI interface planarity (Å) by interface segmentation. ANOVA p-value < 0.05.

| Segmentation | ANOVA Group | Mean | SD | N | Min | Med | Max | Mode |
| --- | --- | --- | --- | --- | --- | --- | --- | --- |
| Single | a | 4.54 | 1.82 | 10694 | 0.40 | 4.20 | 19.86 | 0.40 |
| Multi | b | 6.28 | 2.80 | 44415 | 0.51 | 5.53 | 20.03 | 0.51 |

**Table S3** - Summary statistics and ANOVA analysis of PPI interface planarity (Å) by interface type. ANOVA p-value < 0.05.

| Interface type | ANOVA Group | Mean | SD | N | Min | Med | Max | Mode |
| --- | --- | --- | --- | --- | --- | --- | --- | --- |
| Enzyme-peptide | a | 4.18 | 0.99 | 822 | 1.91 | 4.07 | 8.17 | 1.91 |
| Protein-peptide | a | 4.40 | 1.21 | 1702 | 0.70 | 4.32 | 9.51 | 0.69 |
| Non-identical | b | 5.74 | 2.65 | 28165 | 1.02 | 5.06 | 20.03 | 1.01 |
| Identical-symmetric | c | 6.52 | 2.72 | 15920 | 0.67 | 5.86 | 19.97 | 0.67 |
| Identical-nonsymmetric | d | 6.00 | 3.03 | 8580 | 0.40 | 5.12 | 19.97 | 0.40 |

**Table S4** - Summary statistics and ANOVA analysis of proportions of secondary structure types in PPI interfaces in interface cores by interface type. Anova p-value < 0.05.

| Segmentation | SST | Side | ANOVA Group | Mean | SD | N | Min | Med | Max | Mode |
| --- | --- | --- | --- | --- | --- | --- | --- | --- | --- | --- |
| Single | Helices | Smaller | a | 0.34 | 0.38 | 10694 | 0.00 | 0.20 | 1.00 | 0.00 |
| Single | Sheets | Smaller | b | 0.08 | 0.17 | 10694 | 0.00 | 0.00 | 1.00 | 0.00 |
| Single | Loops | Smaller | c | 0.58 | 0.35 | 10694 | 0.00 | 0.60 | 1.00 | 1.00 |
| Multi | Helices | Smaller | a | 0.35 | 0.28 | 44415 | 0.00 | 0.32 | 1.00 | 0.00 |
| Multi | Sheets | Smaller | d | 0.16 | 0.20 | 10694 | 0.00 | 0.09 | 1.00 | 0.00 |
| Multi | Loops | Smaller | e | 0.49 | 0.23 | 10694 | 0.00 | 0.47 | 1.00 | 0.50 |
| Single | Helices | Larger | f | 0.41 | 0.34 | 10694 | 0.00 | 0.38 | 1.00 | 0.00 |
| Single | Sheets | Larger | g | 0.18 | 0.23 | 10694 | 0.00 | 0.07 | 1.00 | 0.00 |
| Single | Loops | Larger | f | 0.41 | 0.27 | 10694 | 0.00 | 0.38 | 1.00 | 0.00 |
| Multi | Helices | Larger | a | 0.35 | 0.27 | 44415 | 0.00 | 0.33 | 1.00 | 0.00 |
| Multi | Sheets | Larger | d | 0.18 | 0.20 | 44415 | 0.00 | 0.11 | 1.00 | 0.00 |
| Multi | Loops | Larger | g | 0.47 | 0.22 | 44415 | 0.00 | 0.46 | 1.00 | 0.50 |

**Table S5** - Summary statistics and ANOVA analysis of proportions of secondary structure types in PPI interfaces in interface periphery by interface type. Anova p-value < 0.05.

| Segmentation | SST | Side | ANOVA Group | Mean | SD | N | Min | Med | Max | Mode |
| --- | --- | --- | --- | --- | --- | --- | --- | --- | --- | --- |
| Single | Helices | Smaller | a | 0.33 | 0.42 | 10694 | 0.00 | 0.00 | 1.00 | 0.00 |
| Single | Sheets | Smaller | b | 0.02 | 0.13 | 10694 | 0.00 | 0.00 | 1.00 | 0.00 |
| Single | Loops | Smaller | a | 0.34 | 0.42 | 10694 | 0.00 | 0.00 | 1.00 | 0.00 |
| Multi | Helices | Smaller | c | 0.36 | 0.32 | 44415 | 0.00 | 0.33 | 1.00 | 0.00 |
| Multi | Sheets | Smaller | d | 0.16 | 0.23 | 10694 | 0.00 | 0.00 | 1.00 | 0.00 |
| Multi | Loops | Smaller | e | 0.46 | 0.29 | 10694 | 0.00 | 0.46 | 1.00 | 0.50 |
| Single | Helices | Larger | f | 0.42 | 0.38 | 10694 | 0.00 | 0.40 | 1.00 | 0.00 |
| Single | Sheets | Larger | dg | 0.16 | 0.26 | 10694 | 0.00 | 0.00 | 1.00 | 0.00 |
| Single | Loops | Larger | h | 0.39 | 0.34 | 10694 | 0.00 | 0.33 | 1.00 | 0.00 |
| Multi | Helices | Larger | i | 0.37 | 0.32 | 44415 | 0.00 | 0.33 | 1.00 | 0.00 |
| Multi | Sheets | Larger | g | 0.17 | 0.24 | 44415 | 0.00 | 0.00 | 1.00 | 0.00 |
| Multi | Loops | Larger | j | 0.45 | 0.29 | 44415 | 0.00 | 0.43 | 1.00 | 0.00 |

**Table S6** - Summary statistics and ANOVA analysis of PPI interface NIP by interface segmentation. ANOVA p-value < 0.05.

| Segmentation | ANOVA Group | Mean | SD | N | Min | Med | Max | Mode |
| --- | --- | --- | --- | --- | --- | --- | --- | --- |
| Single | a | 0.00078 | 0.00057 | 10694 | 0.00002 | 0.000628 | 0.00918 | 0.000265 |
| Multi | b | 0.0004 | 0.00041 | 44415 | 0 | 0.000256 | 0.01391 | 0.000081 |

**Table S7** - Summary statistics and ANOVA analysis of PPI interface NIP by interface type. ANOVA p-value < 0.05.

| Interface type | ANOVA Group | Mean | SD | N | Min | Med | Max | Mode |
| --- | --- | --- | --- | --- | --- | --- | --- | --- |
| Enzyme-peptide | a | 0.0008 | 0.00036 | 822 | 0.00018 | 0.000735 | 0.00237 | 0.000336 |
| Protein-peptide | a | 0.00078 | 0.00055 | 1702 | 0.00018 | 0.00064 | 0.00918 | 0.000242 |
| Non-identical | b | 0.00053 | 0.00048 | 28165 | 0 | 0.000373 | 0.00403 | 0.000009 |
| Identical-symmetric | c | 0.0003 | 0.00037 | 15920 | 0 | 0.000172 | 0.00443 | 0.000099 |
| Identical-nonsymmetric | b | 0.00052 | 0.00049 | 8580 | 0 | 0.00037 | 0.01391 | 0.000156 |

**Table S8** - Summary statistics and ANOVA analysis of PPI interface NSc by interface segmentation. ANOVA p-value < 0.05.

| Segmentation | ANOVA Group | Mean | SD | N | Min | Med | Max | Mode |
| --- | --- | --- | --- | --- | --- | --- | --- | --- |
| Single | a | 0.00093 | 0.00063 | 10694 | 0 | 0.0007755 | 0.0071 | 0.000526 |
| Multi | b | 0.00051 | 0.00048 | 44415 | 0 | 0.000376 | 0.00864 | 0.000154 |

**Table S9** - Summary statistics and ANOVA analysis of PPI interface NSc by interface type. ANOVA p-value < 0.05.

| Interface type | ANOVA Group | Mean | SD | N | Min | Med | Max | Mode |
| --- | --- | --- | --- | --- | --- | --- | --- | --- |
| Enzyme-peptide | a | 0.00124 | 0.00056 | 822 | 0.00027 | 0.0011165 | 0.00396 | 0.000551 |
| Protein-peptide | b | 0.00104 | 0.00054 | 1702 | 0.00033 | 0.000902 | 0.00467 | 0.000558 |
| Non-identical | c | 0.00065 | 0.00055 | 28165 | 0 | 0.000504 | 0.00864 | 0.000154 |
| Identical-symmetric | d | 0.00038 | 0.00039 | 15920 | 0 | 0.000243 | 0.00701 | 0.000159 |
| Identical-nonsymmetric | c | 0.00065 | 0.00055 | 8580 | 0 | 0.000509 | 0.00572 | 0.000143 |

**Table S10** - Summary statistics and ANOVA analysis of PPI interface BSA by interface segmentation. ANOVA p-value < 0.05.

| Segmentation | ANOVA Group | Mean | SD | N | Min | Med | Max | Mode |
| --- | --- | --- | --- | --- | --- | --- | --- | --- |
| Single | a | 1173.17 | 957.48 | 10694 | 195.54 | 904.76 | 13496.83 | 386.22 |
| Multi | b | 2259.09 | 2259.79 | 44415 | 152.55 | 1621.27 | 27463.39 | 316.977 |

**Table S11** - Summary statistics and ANOVA analysis of PPI interface BSA by interface type. ANOVA p-value < 0.05.

| Interface type | ANOVA Group | Mean | SD | N | Min | Med | Max | Mode |
| --- | --- | --- | --- | --- | --- | --- | --- | --- |
| Enzyme-peptide | a | 828.08 | 294.95 | 822 | 315.57 | 772.13 | 2073.21 | 315.574 |
| Protein-peptide | a | 1005.93 | 455.06 | 1702 | 268.02 | 913.98 | 2403.32 | 268.024 |
| Non-identical | b | 1819.32 | 2053.82 | 28165 | 152.55 | 1253.41 | 22558.12 | 316.97 |
| Identical-symmetric | c | 2707.22 | 2030.73 | 15920 | 155.22 | 2230.63 | 26718.4 | 816.413 |
| Identical-nonsymmetric | d | 1900.42 | 2429.5 | 8580 | 199.08 | 1192.15 | 27463.39 | 318.71 |

**Table S12 - Summary statistics and ANOVA analysis of PPI interface proportion of core residues by interface segmentation.** ANOVA p-value < 0.05. Larger side refers to the side of the pairwise interface with fewer interacting residues than the smaller side.

| Segmentation | Interface side | ANOVA Group | Mean | SD | N | Min | Med | Max | Mode |
| --- | --- | --- | --- | --- | --- | --- | --- | --- | --- |
| Single | Larger | a | 0.23 | 0.16 | 10694 | 0 | 0.19 | 0.94 | 0 |
| Single | Smaller | b | 0.1 | 0.14 | 10694 | 0 | 0.04 | 1 | 0 |
| Multi | Larger | c | 0.2 | 0.14 | 44415 | 0 | 0.21 | 0.62 | 0 |
| Multi | Smaller | d | 0.19 | 0.15 | 44415 | 0 | 0.19 | 0.77 | 0 |

**Table S13 - Summary statistics and ANOVA analysis of PPI interface proportion of core residues by interface type.** ANOVA p-value < 0.05. Larger side refers to the side of the pairwise interface with fewer interacting residues than the smaller side.

| Interface type | Interface side | ANOVA Group | Mean | SD | N | Min | Med | Max | Mode |
| --- | --- | --- | --- | --- | --- | --- | --- | --- | --- |
| Enzyme-peptide | Larger | a | 0.29 | 0.14 | 822 | 0.00 | 0.29 | 0.74 | 0.33 |
| Enzyme-peptide | Smaller | b | 0.09 | 0.14 | 822 | 0.00 | 0.00 | 0.67 | 0.00 |
| Protein-peptide | Larger | a | 0.30 | 0.18 | 1702 | 0.00 | 0.29 | 0.94 | 0.00 |
| Protein-peptide | Smaller | c | 0.15 | 0.19 | 1702 | 0.00 | 0.10 | 1.00 | 0.00 |
| Non-identical | Larger | d | 0.19 | 0.14 | 28124 | 0.00 | 0.18 | 0.64 | 0.00 |
| Non-identical | Smaller | c | 0.15 | 0.14 | 28124 | 0.00 | 0.13 | 0.82 | 0.00 |
| Identical-symmetric | Larger | e | 0.25 | 0.14 | 15912 | 0.00 | 0.27 | 0.62 | 0.00 |
| Identical-symmetric | Smaller | e | 0.26 | 0.14 | 15912 | 0.00 | 0.27 | 0.62 | 0.00 |
| Identical-nonsymmetric | Larger | f | 0.17 | 0.13 | 8549 | 0.00 | 0.17 | 0.62 | 0.00 |
| Identical-nonsymmetric | Smaller | c | 0.15 | 0.14 | 8549 | 0.00 | 0.14 | 0.61 | 0.00 |

**Table S14** - Summary statistics and ANOVA analysis of VdW Clash interactions at PPI interfaces by interface segmentation. ANOVA p-value < 0.05.

| Segmentation | ANOVA Group | Mean | SD | N | Min | Med | Max | Mode |
| --- | --- | --- | --- | --- | --- | --- | --- | --- |
| Single | a | 1.53 | 0.43 | 10694 | -0.62 | 1.52 | 2.97 | 1.00 |
| Multi | b | 1.52 | 0.40 | 44415 | -1.17 | 1.48 | 3.52 | 1.00 |

**Table S15** - Summary statistics and ANOVA analysis of VdW Clash interactions at PPI interfaces by interface type. ANOVA p-value < 0.05.

| Interface type | ANOVA Group | Mean | SD | N | Min | Med | Max | Mode |
| --- | --- | --- | --- | --- | --- | --- | --- | --- |
| Enzyme-peptide | a | 1.57 | 0.32 | 822 | 0.71 | 1.57 | 2.51 | 0.70 |
| Protein-peptide | b | 1.47 | 0.30 | 1702 | 0.20 | 1.46 | 2.73 | 0.19 |
| Non-identical | a | 1.56 | 0.42 | 28165 | -1.13 | 1.53 | 3.52 | 1.00 |
| Identical-symmetric | c | 1.42 | 0.37 | 15920 | -1.17 | 1.39 | 2.97 | 1.00 |
| Identical-nonsymmetric | a | 1.58 | 0.43 | 8580 | -1.16 | 1.57 | 2.96 | 1.00 |

**Table S16** - Summary statistics and ANOVA analysis of VdW interactions at PPI interfaces by interface segmentation. ANOVA p-value < 0.05.

| Segmentation | ANOVA Group | Mean | SD | N | Min | Med | Max | Mode |
| --- | --- | --- | --- | --- | --- | --- | --- | --- |
| Single | a | 1.27 | 0.43 | 10694 | -0.82 | 1.26 | 2.76 | 1.00 |
| Multi | b | 1.26 | 0.39 | 44415 | -1.31 | 1.22 | 3.03 | 0.00 |

**Table S17** - Summary statistics and ANOVA analysis of VdW interactions at PPI interfaces by interface type. ANOVA p-value < 0.05.

| Interface type | ANOVA Group | Mean | SD | N | Min | Med | Max | Mode |
| --- | --- | --- | --- | --- | --- | --- | --- | --- |
| Enzyme-peptide | a | 1.29 | 0.33 | 822 | 0.41 | 1.29 | 2.29 | 0.40 |
| Protein-peptide | b | 1.18 | 0.30 | 1702 | 0.15 | 1.16 | 2.41 | 0.14 |
| Non-identical | a | 1.30 | 0.41 | 28165 | -1.31 | 1.28 | 3.03 | 0.00 |
| Identical-symmetric | b | 1.16 | 0.37 | 15920 | -1.07 | 1.13 | 2.76 | 0.00 |
| Identical-nonsymmetric | c | 1.32 | 0.42 | 8580 | -1.24 | 1.30 | 2.61 | 0.00 |

**Table S18** - Summary statistics and ANOVA analysis of Proximal interactions at PPI interfaces by interface segmentation. ANOVA p-value < 0.05.

| Segmentation | ANOVA Group | Mean | SD | N | Min | Med | Max | Mode |
| --- | --- | --- | --- | --- | --- | --- | --- | --- |
| Single | a | 2.99 | 0.43 | 10694 | 1.00 | 2.99 | 4.38 | 1.00 |
| Multi | a | 2.99 | 0.38 | 44415 | 1.00 | 2.96 | 4.55 | 1.00 |

**Table S19** - Summary statistics and ANOVA analysis of Proximal interactions at PPI interfaces by interface type. ANOVA p-value < 0.05.

| Interface type | ANOVA Group | Mean | SD | N | Min | Med | Max | Mode |
| --- | --- | --- | --- | --- | --- | --- | --- | --- |
| Enzyme-peptide | a | 3.06 | 0.31 | 822 | 2.22 | 3.07 | 3.99 | 2.21 |
| Protein-peptide | b | 2.96 | 0.29 | 1702 | 2.12 | 2.95 | 4.12 | 2.11 |
| Non-identical | c | 3.02 | 0.39 | 28165 | 1.00 | 3.01 | 4.52 | 1.00 |
| Identical-symmetric | d | 2.90 | 0.37 | 15920 | 1.00 | 2.88 | 4.55 | 1.00 |
| Identical-nonsymmetric | a | 3.05 | 0.42 | 8580 | 1.00 | 3.04 | 4.33 | 1.00 |

**Table S20** - Summary statistics and ANOVA analysis of Hydrogen bonds at PPI interfaces by interface segmentation. ANOVA p-value < 0.05.

| Segmentation | ANOVA Group | Mean | SD | N | Min | Med | Max | Mode |
| --- | --- | --- | --- | --- | --- | --- | --- | --- |
| Single | a | 1.45 | 0.42 | 10694 | -0.14 | 1.45 | 2.83 | 0.00 |
| Multi | b | 1.44 | 0.38 | 44415 | 0.00 | 1.41 | 2.96 | 0.00 |

**Table S21** - Summary statistics and ANOVA analysis of Hydrogen bonds at PPI interfaces by interface type. ANOVA p-value < 0.05.

| Interface type | ANOVA Group | Mean | SD | N | Min | Med | Max | Mode |
| --- | --- | --- | --- | --- | --- | --- | --- | --- |
| Enzyme-peptide | a,b | 1.49 | 0.32 | 822 | 0.62 | 1.51 | 2.42 | 0.62 |
| Protein-peptide | c | 1.39 | 0.29 | 1702 | 0.20 | 1.38 | 2.56 | 0.19 |
| Non-identical | b | 1.47 | 0.40 | 28165 | 0.00 | 1.46 | 2.96 | 0.00 |
| Identical-symmetric | d | 1.36 | 0.36 | 15920 | -0.14 | 1.33 | 2.96 | 0.00 |
| Identical-nonsymmetric | a | 1.50 | 0.42 | 8580 | 0.00 | 1.50 | 2.77 | 0.00 |

**Table S22** - Summary statistics and ANOVA analysis of Weak Hydrogen bonds at PPI interfaces by interface segmentation. ANOVA p-value < 0.05.

| Segmentation | ANOVA Group | Mean | SD | N | Min | Med | Max | Mode |
| --- | --- | --- | --- | --- | --- | --- | --- | --- |
| Single | a | 1.30 | 0.42 | 10694 | -0.88 | 1.30 | 2.69 | 0.00 |
| Multi | a | 1.29 | 0.38 | 44415 | -1.02 | 1.27 | 2.84 | 0.00 |

**Table S23** - Summary statistics and ANOVA analysis of Weak Hydrogen bonds at PPI interfaces by interface type. ANOVA p-value < 0.05.

| Interface type | ANOVA Group | Mean | SD | N | Min | Med | Max | Mode |
| --- | --- | --- | --- | --- | --- | --- | --- | --- |
| Enzyme-peptide | a | 1.37 | 0.31 | 822 | 0.41 | 1.37 | 2.28 | 0.40 |
| Protein-peptide | b | 1.27 | 0.29 | 1702 | 0.26 | 1.27 | 2.38 | 0.25 |
| Non-identical | c | 1.31 | 0.39 | 28165 | -1.02 | 1.30 | 2.82 | 0.00 |
| Identical-symmetric | d | 1.22 | 0.36 | 15920 | -0.34 | 1.20 | 2.84 | 0.00 |
| Identical-nonsymmetric | a | 1.35 | 0.42 | 8580 | -0.93 | 1.35 | 2.64 | 0.00 |

**Table S24** - Summary statistics and ANOVA analysis of Ionic interactions at PPI interfaces by interface segmentation. ANOVA p-value < 0.05.

| Segmentation | ANOVA Group | Mean | SD | N | Min | Med | Max | Mode |
| --- | --- | --- | --- | --- | --- | --- | --- | --- |
| Single | a | 0.40 | 0.50 | 10694 | -1.84 | 0.43 | 2.09 | 0.00 |
| Multi | b | 0.44 | 0.45 | 44415 | -1.77 | 0.44 | 2.31 | 0.00 |

**Table S25** - Summary statistics and ANOVA analysis of Ionic interactions at PPI interfaces by interface type. ANOVA p-value < 0.05.

| Interface type | ANOVA Group | Mean | SD | N | Min | Med | Max | Mode |
| --- | --- | --- | --- | --- | --- | --- | --- | --- |
| Enzyme-peptide | a | 0.53 | 0.43 | 822 | -0.99 | 0.57 | 1.62 | 0.00 |
| Protein-peptide | b | 0.45 | 0.35 | 1702 | -1.14 | 0.50 | 1.73 | 0.00 |
| Non-identical | c | 0.41 | 0.45 | 28165 | -1.84 | 0.41 | 1.99 | 0.00 |
| Identical-symmetric | b | 0.43 | 0.44 | 15920 | -1.77 | 0.44 | 2.31 | 0.00 |
| Identical-nonsymmetric | a | 0.50 | 0.51 | 8580 | -1.54 | 0.52 | 2.01 | 0.00 |

**Table S26** - Summary statistics and ANOVA analysis of Hydrophobic interactions at PPI interfaces by interface segmentation. ANOVA p-value < 0.05.

| Segmentation | ANOVA Group | Mean | SD | N | Min | Med | Max | Mode |
| --- | --- | --- | --- | --- | --- | --- | --- | --- |
| Single | a | 1.71 | 0.49 | 10694 | -0.25 | 1.75 | 3.20 | 0.00 |
| Multi | b | 1.73 | 0.45 | 44415 | -1.05 | 1.74 | 3.42 | 0.00 |

**Table S27** - Summary statistics and ANOVA analysis of Hydrophobic interactions at PPI interfaces by interface type. ANOVA p-value < 0.05.

| Interface type | ANOVA Group | Mean | SD | N | Min | Med | Max | Mode |
| --- | --- | --- | --- | --- | --- | --- | --- | --- |
| Enzyme-peptide | a | 1.87 | 0.32 | 822 | 0.78 | 1.88 | 2.81 | 0.78 |
| Protein-peptide | b | 1.76 | 0.29 | 1702 | 0.69 | 1.77 | 2.88 | 0.68 |
| Non-identical | c | 1.72 | 0.48 | 28165 | -1.05 | 1.75 | 3.27 | 0.00 |
| Identical-symmetric | d | 1.70 | 0.40 | 15920 | -0.40 | 1.69 | 3.42 | 0.00 |
| Identical-nonsymmetric | b | 1.79 | 0.51 | 8580 | -0.69 | 1.82 | 3.16 | 0.00 |

**Table S28** - Summary statistics and ANOVA analysis of Carbonyl interactions at PPI interfaces by interface segmentation. ANOVA p-value < 0.05.

| Segmentation | ANOVA Group | Mean | SD | N | Min | Med | Max | Mode |
| --- | --- | --- | --- | --- | --- | --- | --- | --- |
| Single | a | 0.44 | 0.54 | 10694 | -1.65 | 0.48 | 2.07 | 0.00 |
| Multi | b | 0.48 | 0.45 | 44415 | -1.66 | 0.46 | 2.53 | 0.00 |

**Table S29** - Summary statistics and ANOVA analysis of Carbonyl interactions at PPI interfaces by interface type. ANOVA p-value < 0.05.

| Interface type | ANOVA Group | Mean | SD | N | Min | Med | Max | Mode |
| --- | --- | --- | --- | --- | --- | --- | --- | --- |
| Enzyme-peptide | a | 0.53 | 0.34 | 822 | -0.63 | 0.54 | 1.36 | -0.63 |
| Protein-peptide | b | 0.47 | 0.38 | 1702 | -1.21 | 0.47 | 1.58 | 0.00 |
| Non-identical | a | 0.52 | 0.48 | 28165 | -1.57 | 0.52 | 2.53 | 0.00 |
| Identical-symmetric | c | 0.35 | 0.44 | 15920 | -1.66 | 0.35 | 2.10 | 0.00 |
| Identical-nonsymmetric | a | 0.54 | 0.48 | 8580 | -1.50 | 0.54 | 2.20 | 0.00 |

**Table S30** - Summary statistics and ANOVA analysis of Polar interactions at PPI interfaces by interface segmentation. ANOVA p-value < 0.05.

| Segmentation | ANOVA Group | Mean | SD | N | Min | Med | Max | Mode |
| --- | --- | --- | --- | --- | --- | --- | --- | --- |
| Single | a | 1.63 | 0.42 | 10694 | 0.00 | 1.62 | 3.03 | 0.00 |
| Multi | b | 1.61 | 0.39 | 44415 | 0.00 | 1.58 | 3.17 | 0.00 |

**Table S31** - Summary statistics and ANOVA analysis of Polar interactions at PPI interfaces by interface type. ANOVA p-value < 0.05.

| Interface type | ANOVA Group | Mean | SD | N | Min | Med | Max | Mode |
| --- | --- | --- | --- | --- | --- | --- | --- | --- |
| Enzyme-peptide | ac | 1.66 | 0.33 | 822 | 0.75 | 1.68 | 2.62 | 0.74 |
| Protein-peptide | b | 1.55 | 0.30 | 1702 | 0.39 | 1.54 | 2.77 | 0.39 |
| Non-identical | c | 1.64 | 0.41 | 28165 | 0.00 | 1.63 | 3.17 | 0.00 |
| Identical-symmetric | b | 1.54 | 0.37 | 15920 | 0.00 | 1.51 | 3.07 | 0.00 |
| Identical-nonsymmetric | a | 1.67 | 0.43 | 8580 | 0.00 | 1.67 | 2.98 | 0.00 |

**Table S32** - Summary statistics and ANOVA analysis of Weak Polar interactions at PPI interfaces by interface segmentation. ANOVA p-value < 0.05.

| Segmentation | ANOVA Group | Mean | SD | N | Min | Med | Max | Mode |
| --- | --- | --- | --- | --- | --- | --- | --- | --- |
| Single | a | 1.40 | 0.43 | 10694 | -0.04 | 1.39 | 2.90 | 0.00 |
| Multi | b | 1.39 | 0.39 | 44415 | 0.00 | 1.35 | 3.23 | 0.00 |

**Table S33** - Summary statistics and ANOVA analysis of Weak Polar interactions at PPI interfaces by interface type. ANOVA p-value < 0.05.

| Interface type | ANOVA Group | Mean | SD | N | Min | Med | Max | Mode |
| --- | --- | --- | --- | --- | --- | --- | --- | --- |
| Enzyme-peptide | ac | 1.43 | 0.32 | 822 | 0.61 | 1.45 | 2.40 | 0.61 |
| Protein-peptide | b | 1.34 | 0.29 | 1702 | -0.04 | 1.34 | 2.53 | -0.04 |
| Non-identical | c | 1.43 | 0.41 | 28165 | 0.00 | 1.42 | 3.23 | 0.00 |
| Identical-symmetric | d | 1.29 | 0.37 | 15920 | 0.00 | 1.27 | 2.90 | 0.00 |
| Identical-nonsymmetric | e | 1.44 | 0.42 | 8580 | 0.00 | 1.44 | 2.74 | 0.00 |

**Table S34** - Summary statistics and ANOVA analysis of atom-ring Carbon-𝜋 interactions at PPI interfaces by interface segmentation. ANOVA p-value < 0.05.

| Segmentation | ANOVA Group | Mean | SD | N | Min | Med | Max | Mode |
| --- | --- | --- | --- | --- | --- | --- | --- | --- |
| Single | a | 0.50 | 0.42 | 10694 | -0.65 | 0.52 | 2.43 | 0.00 |
| Multi | b | 0.55 | 0.42 | 44415 | -0.90 | 0.57 | 2.54 | 0.00 |

**Table S35** - Summary statistics and ANOVA analysis of atom-ring Carbon-𝜋 interactions at PPI interfaces by interface type. ANOVA p-value < 0.05.

| Interface type | ANOVA Group | Mean | SD | N | Min | Med | Max | Mode |
| --- | --- | --- | --- | --- | --- | --- | --- | --- |
| Enzyme-peptide | a | 0.69 | 0.38 | 822 | -0.19 | 0.77 | 1.99 | 0.00 |
| Protein-peptide | a | 0.73 | 0.37 | 1702 | -0.17 | 0.80 | 1.86 | 0.00 |
| Non-identical | b | 0.51 | 0.42 | 28165 | -0.77 | 0.52 | 2.34 | 0.00 |
| Identical-symmetric | c | 0.59 | 0.40 | 15920 | -0.90 | 0.63 | 2.30 | 0.00 |
| Identical-nonsymmetric | b | 0.50 | 0.44 | 8580 | -0.79 | 0.49 | 2.54 | 0.00 |

**Table S36** - Summary statistics and ANOVA analysis of atom-ring Cation-𝜋 interactions at PPI interfaces by interface segmentation. ANOVA p-value < 0.05.

| Segmentation | ANOVA Group | Mean | SD | N | Min | Med | Max | Mode |
| --- | --- | --- | --- | --- | --- | --- | --- | --- |
| Single | a | 0.03 | 0.18 | 10694 | -1.00 | 0.00 | 1.45 | 0.00 |
| Multi | b | 0.00 | 0.23 | 44415 | -1.33 | 0.00 | 1.43 | 0.00 |

**Table S37** - Summary statistics and ANOVA analysis of atom-ring Cation-𝜋 interactions at PPI interfaces by interface type. ANOVA p-value < 0.05.

| Interface type | ANOVA Group | Mean | SD | N | Min | Med | Max | Mode |
| --- | --- | --- | --- | --- | --- | --- | --- | --- |
| Enzyme-peptide | a | 0.06 | 0.17 | 822 | -0.30 | 0.00 | 0.98 | 0.00 |
| Protein-peptide | a | 0.07 | 0.21 | 1702 | -0.33 | 0.00 | 1.31 | 0.00 |
| Non-identical | b | 0.01 | 0.21 | 28165 | -1.28 | 0.00 | 1.43 | 0.00 |
| Identical-symmetric | c | 0.00 | 0.22 | 15920 | -1.15 | 0.00 | 1.43 | 0.00 |
| Identical-nonsymmetric | b | 0.01 | 0.23 | 8580 | -1.33 | 0.00 | 1.45 | 0.00 |

**Table S38** - Summary statistics and ANOVA analysis of atom-ring Donor-𝜋 interactions at PPI interfaces by interface segmentation. ANOVA p-value < 0.05.

| Segmentation | ANOVA Group | Mean | SD | N | Min | Med | Max | Mode |
| --- | --- | --- | --- | --- | --- | --- | --- | --- |
| Single | a | 0.09 | 0.25 | 10694 | -1.12 | 0.00 | 1.62 | 0.00 |
| Multi | b | 0.07 | 0.29 | 44415 | -1.26 | 0.00 | 1.72 | 0.00 |

**Table S39** - Summary statistics and ANOVA analysis of atom-ring Donor-𝜋 interactions at PPI interfaces by interface type. ANOVA p-value < 0.05.

| Interface type | ANOVA Group | Mean | SD | N | Min | Med | Max | Mode |
| --- | --- | --- | --- | --- | --- | --- | --- | --- |
| Enzyme-peptide | a | 0.18 | 0.25 | 822 | -0.20 | 0.00 | 1.60 | 0.00 |
| Protein-peptide | a | 0.17 | 0.26 | 1702 | -0.32 | 0.02 | 1.35 | 0.00 |
| Non-identical | b | 0.07 | 0.28 | 28165 | -1.26 | 0.00 | 1.72 | 0.00 |
| Identical-symmetric | c | 0.06 | 0.29 | 15920 | -1.12 | 0.00 | 1.60 | 0.00 |
| Identical-nonsymmetric | b | 0.07 | 0.29 | 8580 | -1.01 | 0.00 | 1.66 | 0.00 |

**Table S40** - Summary statistics and ANOVA analysis of Ring-Ring interactions at PPI interfaces by interface segmentation. ANOVA p-value < 0.05.

| Segmentation | ANOVA Group | Mean | SD | N | Min | Med | Max | Mode |
| --- | --- | --- | --- | --- | --- | --- | --- | --- |
| Single | a | 0.27 | 0.49 | 10694 | -1.83 | 0.27 | 1.91 | 0.00 |
| Multi | b | 0.26 | 0.48 | 44415 | -1.87 | 0.24 | 2.22 | 0.00 |

**Table S41** - Summary statistics and ANOVA analysis of Ring-Ring interactions at PPI interfaces by interface type. ANOVA p-value < 0.05.

| Interface type | ANOVA Group | Mean | SD | N | Min | Med | Max | Mode |
| --- | --- | --- | --- | --- | --- | --- | --- | --- |
| Enzyme-peptide | a | 0.40 | 0.47 | 822 | -0.75 | 0.43 | 1.52 | 0.00 |
| Protein-peptide | b | 0.33 | 0.39 | 1702 | -0.99 | 0.42 | 1.46 | 0.00 |
| Non-identical | c | 0.28 | 0.47 | 28165 | -1.57 | 0.27 | 1.91 | 0.00 |
| Identical-symmetric | d | 0.19 | 0.48 | 15920 | -1.87 | 0.17 | 2.22 | 0.00 |
| Identical-nonsymmetric | b | 0.31 | 0.52 | 8580 | -1.68 | 0.28 | 1.99 | 0.00 |

**Table S42** - Summary statistics and ANOVA analysis of Amide-Amide interactions at PPI interfaces by interface segmentation. ANOVA p-value < 0.05.

| Segmentation | ANOVA Group | Mean | SD | N | Min | Med | Max | Mode |
| --- | --- | --- | --- | --- | --- | --- | --- | --- |
| Single | a | 0.12 | 0.51 | 10694 | -1.71 | 0.13 | 1.73 | 0.00 |
| Multi | b | 0.15 | 0.43 | 44415 | -1.71 | 0.12 | 1.91 | 0.00 |

**Table S43** - Summary statistics and ANOVA analysis of Amide-Amide interactions at PPI interfaces by interface type. ANOVA p-value < 0.05.

| Interface type | ANOVA Group | Mean | SD | N | Min | Med | Max | Mode |
| --- | --- | --- | --- | --- | --- | --- | --- | --- |
| Enzyme-peptide | a | 0.15 | 0.41 | 822 | -1.05 | 0.20 | 1.29 | 0.00 |
| Protein-peptide | b | 0.00 | 0.41 | 1702 | -1.26 | 0.00 | 1.27 | 0.00 |
| Non-identical | c | 0.20 | 0.45 | 28165 | -1.53 | 0.19 | 1.91 | 0.00 |
| Identical-symmetric | b | 0.03 | 0.42 | 15920 | -1.71 | 0.02 | 1.83 | 0.00 |
| Identical-nonsymmetric | d | 0.23 | 0.47 | 8580 | -1.73 | 0.19 | 1.85 | 0.00 |

**Table S44** - Summary statistics and ANOVA analysis of Amide-Ring interactions at PPI interfaces by interface segmentation. ANOVA p-value < 0.05.

| Segmentation | ANOVA Group | Mean | SD | N | Min | Med | Max | Mode |
| --- | --- | --- | --- | --- | --- | --- | --- | --- |
| Single | a | 0.74 | 0.52 | 10694 | -1.12 | 0.79 | 2.28 | 0.00 |
| Multi | b | 0.75 | 0.47 | 44415 | -1.04 | 0.75 | 2.60 | 0.00 |

**Table S45** - Summary statistics and ANOVA analysis of Amide-Ring interactions at PPI interfaces by interface type. ANOVA p-value < 0.05.

| Interface type | ANOVA Group | Mean | SD | N | Min | Med | Max | Mode |
| --- | --- | --- | --- | --- | --- | --- | --- | --- |
| Enzyme-peptide | a | 0.91 | 0.43 | 822 | -0.15 | 0.95 | 1.89 | 0.00 |
| Protein-peptide | b | 0.84 | 0.40 | 1702 | -0.18 | 0.88 | 1.86 | 0.00 |
| Non-identical | c | 0.77 | 0.48 | 28165 | -0.92 | 0.79 | 2.29 | 0.00 |
| Identical-symmetric | d | 0.67 | 0.46 | 15920 | -1.12 | 0.68 | 2.60 | 0.00 |
| Identical-nonsymmetric | e | 0.79 | 0.51 | 8580 | -1.04 | 0.81 | 2.40 | 0.00 |

**Table S46** - Summary statistics and ANOVA analysis of PPI interface use of concavity at their deepest and average depth by interface segmentation. ANOVA p-value < 0.05.

| Segmentation | Concavity | ANOVA Group | Mean | SD | N | Min | Med | Max | Mode |
| --- | --- | --- | --- | --- | --- | --- | --- | --- | --- |
| Single | average | a | 4.37 | 1.34 | 10694 | 1.04 | 4.30 | 9.65 | 5.13 |
| Single | deepest | b | 0.81 | 0.84 | 10694 | 0.35 | 0.71 | 8.81 | 0.58 |
| Multi | average | c | 4.40 | 0.71 | 44415 | 1.77 | 4.27 | 9.13 | 4.10 |
| Multi | deepest | d | 1.03 | 0.54 | 44415 | 0.29 | 0.66 | 8.77 | 0.62 |

**Table S47** - Summary statistics and ANOVA analysis of PPI interface use of concavity at their deepest and average depth by interface type. ANOVA p-value < 0.05.

| Interface type | Concavity | ANOVA Group | Mean | SD | N | Min | Med | Max | Mode |
| --- | --- | --- | --- | --- | --- | --- | --- | --- | --- |
| Enzyme-peptide | average | a | 3.11 | 1.21 | 822 | 1.04 | 2.98 | 9.65 | 1.04 |
| Enzyme-peptide | deepest | b | 0.75 | 0.78 | 822 | 0.45 | 0.61 | 7.76 | 0.58 |
| Protein-peptide | average | c | 3.45 | 1.45 | 1702 | 1.07 | 3.24 | 9.09 | 2.86 |
| Protein-peptide | deepest | d | 0.85 | 0.85 | 1702 | 0.44 | 0.61 | 6.78 | 0.56 |
| Non-identical | average | e | 4.54 | 0.91 | 28124 | 1.60 | 4.39 | 9.13 | 4.10 |
| Non-identical | deepest | f | 0.93 | 0.69 | 28124 | 0.29 | 0.69 | 8.81 | 0.62 |
| Identical-symmetric | average | g | 4.22 | 0.55 | 15912 | 2.86 | 4.13 | 8.07 | 4.32 |
| Identical-symmetric | deepest | b | 0.70 | 0.30 | 15912 | 0.34 | 0.63 | 7.35 | 0.60 |
| Identical-nonsymmetric | average | h | 4.58 | 0.74 | 8549 | 2.11 | 4.44 | 8.43 | 5.13 |
| Identical-nonsymmetric | deepest | d | 0.87 | 0.63 | 8549 | 0.36 | 0.69 | 7.77 | 0.63 |

**Table S48** - Summary statistics and ANOVA analysis of interface length by interface segmentation. ANOVA p-value < 0.05.

| Segmentation | ANOVA Group | Mean | SD | N | Min | Med | Max | Mode |
| --- | --- | --- | --- | --- | --- | --- | --- | --- |
| Single | a | 10.99 | 0.65 | 10694 | 2 | 8 | 238 | 5 |
| Multi | b | 27.21 | 26.84 | 44415 | 3 | 20 | 516 | 5 |

**Table S49** - Summary statistics and ANOVA analysis of interface length by interface segmentation. ANOVA p-value < 0.05.

| Interface type | ANOVA Group | Mean | SD | N | Min | Med | Max | Mode |
| --- | --- | --- | --- | --- | --- | --- | --- | --- |
| Enzyme-peptide | a | 5.32 | 2.05 | 822 | 2 | 5 | 10 | 4 |
| Protein-peptide | a | 6.72 | 2.02 | 1702 | 2 | 7 | 10 | 9 |
| Non-identical | b | 20.69 | 23.34 | 28165 | 3 | 14 | 249 | 5 |
| Identical-symmetric | c | 33.41 | 24.23 | 15920 | 5 | 28 | 343 | 19 |
| Identical-nonsymmetric | d | 23.04 | 30.71 | 8580 | 3 | 14 | 516 | 5 |

**Table S50** - Summary statistics and ANOVA analysis of chain length by interface segmentation. ANOVA p-value < 0.05.

| Segmentation | ANOVA Group | Mean | SD | N | Min | Med | Max | Mode |
| --- | --- | --- | --- | --- | --- | --- | --- | --- |
| Single | a | 107.54 | 164.42 | 10694 | 2 | 47 | 2136 | 9 |
| Multi | b | 284.27 | 236.85 | 44415 | 5 | 222 | 3795 | 213 |

**Table S51** - Summary statistics and ANOVA analysis of chain length by interface segmentation. ANOVA p-value < 0.05.

| Interface type | ANOVA Group | Mean | SD | N | Min | Med | Max | Mode |
| --- | --- | --- | --- | --- | --- | --- | --- | --- |
| Enzyme-peptide | a | 5.76 | 2.32 | 822 | 2 | 6 | 10 | 4 |
| Protein-peptide | a | 7.63 | 2.07 | 1702 | 2 | 8 | 10 | 9 |
| Non-identical | b | 246.89 | 263.23 | 28165 | 11 | 178 | 3795 | 11 |
| Identical-symmetric | c | 277.15 | 173.05 | 15920 | 32 | 250 | 2750 | 141 |
| Identical-nonsymmetric | c | 281.32 | 230.72 | 8580 | 11 | 229 | 3303 | 121 |

**Table S52** - Summary statistics and ANOVA analysis of interface residue use of concavity, by interface segmentation, secondary structure (SST), and solvent accessibility. ANOVA p-value < 0.05.

| Segmentation | Exposure | SST | ANOVA group | Mean | SD | N | Min | Med | Max | Mode |
| --- | --- | --- | --- | --- | --- | --- | --- | --- | --- | --- |
| Single | core | helices | ag | 1.23 | 0.67 | 30698 | 0.45 | 0.98 | 5.55 | 0.75 |
| Single | core | sheets | b | 1.29 | 1.07 | 13539 | 0.44 | 0.91 | 8.42 | 0.65 |
| Single | core | loops | c | 2.10 | 1.68 | 14655 | 0.47 | 1.30 | 8.86 | 0.83 |
| Single | periphery | helices | d | 1.80 | 0.81 | 97394 | 0.41 | 1.64 | 6.62 | 1.43 |
| Single | periphery | sheets | e | 2.38 | 1.62 | 26462 | 0.43 | 1.81 | 8.25 | 0.78 |
| Single | periphery | loops | f | 3.30 | 1.67 | 107417 | 0.42 | 3.22 | 9.88 | 1.33 |
| Multi | core | helices | a | 1.24 | 0.65 | 272412 | 0.38 | 1.01 | 6.01 | 0.74 |
| Multi | core | sheets | g | 1.25 | 0.76 | 158120 | 0.32 | 0.97 | 7.61 | 0.68 |
| Multi | core | loops | h | 1.61 | 0.96 | 211237 | 0.40 | 1.26 | 8.73 | 0.87 |
| Multi | periphery | helices | I | 1.75 | 0.88 | 601464 | 0.39 | 1.53 | 7.34 | 0.88 |
| Multi | periphery | sheets | d | 1.80 | 1.09 | 254394 | 0.37 | 1.42 | 8.18 | 0.72 |
| Multi | periphery | loops | j | 2.57 | 1.35 | 898933 | 0.36 | 2.34 | 9.47 | 1.03 |

**Table S53** - Summary statistics and ANOVA analysis of interface residue use of concavity, by interface type, secondary structure (SST), and solvent accessibility. ANOVA p-value < 0.05.

| Interface type | Exposure | SST | ANOVA group | Mean | SD | N | Min | Med | Max | Mode |
| --- | --- | --- | --- | --- | --- | --- | --- | --- | --- | --- |
| Enzyme-peptide | core | helices | a | 0.79 | 0.35 | 1003 | 0.45 | 0.71 | 4.86 | 0.66 |
| Enzyme-peptide | core | sheets | b,g,p,r | 1.23 | 1.11 | 1294 | 0.51 | 0.89 | 7.36 | 0.65 |
| Enzyme-peptide | core | loops | c,n,o,s | 1.86 | 1.90 | 1683 | 0.51 | 0.94 | 8.79 | 0.82 |
| Enzyme-peptide | periphery | helices | d | 1.40 | 0.88 | 1849 | 0.49 | 1.06 | 5.30 | 0.73 |
| Enzyme-peptide | periphery | sheets | e | 2.41 | 1.91 | 2287 | 0.51 | 1.49 | 7.37 | 0.80 |
| Enzyme-peptide | periphery | loops | f | 3.49 | 2.22 | 7790 | 0.52 | 3.09 | 9.88 | 0.97 |
| Protein-peptide | core | helices | a | 0.87 | 0.41 | 4554 | 0.48 | 0.77 | 5.20 | 0.71 |
| Protein-peptide | core | sheets | g | 1.11 | 1.04 | 3383 | 0.44 | 0.83 | 7.67 | 0.74 |
| Protein-peptide | core | loops | h | 3.05 | 2.27 | 3014 | 0.49 | 2.19 | 8.86 | 0.78 |
| Protein-peptide | periphery | helices | I | 1.54 | 0.83 | 7565 | 0.49 | 1.32 | 6.02 | 0.82 |
| Protein-peptide | periphery | sheets | j | 2.18 | 1.75 | 4091 | 0.50 | 1.31 | 7.51 | 0.74 |
| Protein-peptide | periphery | loops | k | 3.75 | 1.92 | 14942 | 0.49 | 3.96 | 9.28 | 1.02 |
| Non-identical | core | helices | b | 1.24 | 0.66 | 117380 | 0.38 | 1.00 | 6.01 | 0.76 |
| Non-identical | core | sheets | b | 1.22 | 0.78 | 74336 | 0.32 | 0.93 | 8.42 | 0.68 |
| Non-identical | core | loops | l | 1.66 | 1.03 | 98715 | 0.40 | 1.28 | 8.73 | 0.87 |
| Non-identical | periphery | helices | m | 1.79 | 0.90 | 307932 | 0.41 | 1.57 | 6.98 | 0.98 |
| Non-identical | periphery | sheets | n | 1.82 | 1.15 | 126756 | 0.42 | 1.41 | 8.25 | 0.71 |
| Non-identical | periphery | loops | o | 2.72 | 1.42 | 496872 | 0.39 | 2.52 | 9.47 | 1.08 |
| Identical-symmetric | core | helices | p | 1.24 | 0.63 | 146895 | 0.42 | 1.02 | 5.45 | 0.78 |
| Identical-symmetric | core | sheets | b | 1.30 | 0.78 | 69368 | 0.34 | 1.00 | 6.65 | 0.69 |
| Identical-symmetric | core | loops | I | 1.57 | 0.93 | 92179 | 0.42 | 1.23 | 8.72 | 0.86 |
| Identical-symmetric | periphery | helices | q | 1.72 | 0.84 | 271587 | 0.39 | 1.52 | 6.56 | 0.92 |
| Identical-symmetric | periphery | sheets | c | 1.87 | 1.13 | 102161 | 0.47 | 1.47 | 7.05 | 0.72 |
| Identical-symmetric | periphery | loops | e | 2.48 | 1.32 | 331673 | 0.36 | 2.23 | 9.05 | 1.03 |
| Identical-nonsymmetric | core | helices | r | 1.30 | 0.69 | 33278 | 0.44 | 1.06 | 5.55 | 0.73 |
| Identical-nonsymmetric | core | sheets | b | 1.28 | 0.80 | 23278 | 0.47 | 0.98 | 6.52 | 0.66 |
| Identical-nonsymmetric | core | loops | l | 1.64 | 0.97 | 30301 | 0.44 | 1.29 | 7.52 | 0.84 |
| Identical-nonsymmetric | periphery | helices | c | 1.77 | 0.86 | 109925 | 0.45 | 1.56 | 7.34 | 1.38 |
| Identical-nonsymmetric | periphery | sheets | s | 1.88 | 1.14 | 45561 | 0.37 | 1.49 | 7.57 | 0.68 |
| Identical-nonsymmetric | periphery | loops | t | 2.60 | 1.35 | 155073 | 0.39 | 2.39 | 9.29 | 1.08 |

**Table S54** - Summary statistics and ANOVA analysis of hotspots per 100 Å^2^ BSA by interface segmentation. ANOVA p-value < 0.05.

| Segmentation | ANOVA Group | Mean | SD | N | Min | Med | Max | Mode |
| --- | --- | --- | --- | --- | --- | --- | --- | --- |
| Single | a | 0.87 | 0.36 | 10694 | 0 | 0.87 | 3.74 | 0 |
| Multi | b | 0.80 | 0.32 | 44415 | 0 | 0.81 | 3.33 | 0 |

**Table S55** - Summary statistics and ANOVA analysis of hotspots per 100 Å^2^ BSA by interface type. ANOVA p-value < 0.05.

| Interface type | ANOVA Group | Mean | SD | N | Min | Med | Max | Mode |
| --- | --- | --- | --- | --- | --- | --- | --- | --- |
| Enzyme-peptide | a | 0.97 | 0.35 | 822 | 0.17 | 0.95 | 2.25 | 0.17 |
| Protein-peptide | b | 1.04 | 0.33 | 1702 | 0.13 | 1.07 | 2.34 | 0.13 |
| Non-identical | c | 0.77 | 0.33 | 28165 | 0 | 0.77 | 3.49 | 0.00 |
| Identical-symmetric | d | 0.90 | 0.30 | 15920 | 0 | 0.92 | 3.74 | 0.00 |
| Identical-nonsymmetric | e | 0.73 | 0.32 | 8580 | 0 | 0.73 | 3.46 | 0.00 |

**Table S56** – Secondary Structure Types classification based on DSSP.

| SST Class | DSSP code | DSSP description |
| --- | --- | --- |
| ɑ-helix | H | Alpha helix (4-12) |
|  | G | 3-10 helix |
|  | I | Pi helix |
| 𝛽-sheet | B | Isolated beta-bridge |
|  | E | Strand |
| Loop | T | Turn |
|  | S | Bend |
|  | - | None |

**Table S57** - Residue exposure based on distance from interacting chain (s) and change in residue side-chain relative solvent accessibility (RSA) on complexation. A residue is considered to be at the interface if at least one of its atoms is within 5 Å of any of the binding partner’s protein atoms.

| Residue % RSA (Single chain) | Residue % RSA (Whole complex) | Is interface? | Exposure category |
| --- | --- | --- | --- |
| ≤ 7 | Any | Any | Protein Core |
| > 7 | Any | False | Surface exposed |
| > 7 | ≤ 7 | True | Interface Core |
| > 7 | > 7 | True | Interface Periphery |
